# Chronic intermittent hypoxia increases Parkinson's disease susceptibility via PPARα-mediated lipid droplet-mitochondrial dysfunction

**DOI:** 10.7150/thno.122944

**Published:** 2026-01-01

**Authors:** Ming-Rui Zhai, Jie Pan, Zhen-Huan Wu, Yu-Ying He, Kai-Run Zhang, Long Ren, Yu-Rong Wang, Yi-Bing Li, Jun Gao, Lei Xiao, Yue-Hua Liu

**Affiliations:** Department of Orthodontics, Shanghai Stomatological Hospital & School of Stomatology, Shanghai Key Laboratory of Craniomaxillofacial Development and Diseases, State Key Laboratory of Brain Function and Disorders, MOE Frontiers Center for Brain Science, and the Institutes of Brain Science, Fudan University, China.

**Keywords:** obstructive sleep apnea, chronic intermittent hypoxia, Parkinson's disease, lipid droplet, mitochondria, PPARα

## Abstract

**Rationale:** Obstructive sleep apnea (OSA), characterized by chronic intermittent hypoxia (CIH), has emerged as a risk factor for Parkinson's Disease (PD). Yet, whether CIH increases PD susceptibility and the underlying mechanisms remain elusive.

**Methods:** To investigate the impact of CIH on PD susceptibility, we established a series of subtoxic PD models subjected to CIH conditions. We analyzed lipid metabolism, with a particular focus on lipid droplets (LDs), in the pathogenesis of CIH-induced PD. Furthermore, we examined the significance of LD-mitochondrial interactions in mediating aberrant LD accumulation within dopaminergic (DA) neurons and identified the tethering proteins implicated in this process. Additionally, we utilized both systemic and region-specific modulation of the peroxisome proliferator-activated receptor α (PPARα) pathway to assess the neuroprotective potential of restoring LD-mitochondrial coupling in PD models.

**Results:** We revealed that CIH significantly exacerbated nigrostriatal DA neurodegeneration and motor dysfunction in subtoxic PD models. Mechanistically, we identified a PPARα-dependent disruption of Mfn2-Plin5 tethering, which impaired LD-mitochondrial interactions, thereby compromising LD turnover and promoting pathological LD accumulation within DA neurons. Crucially, pharmacological interventions targeting the LD-mitochondrial axis, including strategies to enhance LD catabolism, inhibit mitochondrial fission, or restore LD-mitochondrial tethering, effectively mitigated nigrostriatal DA neurodegeneration in CIH-preconditioned subtoxic PD models.

**Conclusions:** This study reveals a previously unrecognized LD-mitochondrial regulatory axis underlying CIH-associated PD pathology and highlights its potential as a therapeutic target against CIH-accelerated neurodegeneration.

## Introduction

Obstructive sleep apnea (OSA) is a common breathing disorder associated with sleep, marked by repeated episodes of apnea or hypopnea during sleep 1. Its prevalence has steadily increased, affecting approximately 5-15% of the global population, with a particularly high incidence in the elderly 2. Chronic intermittent hypoxia (CIH), a hallmark feature of OSA, has been shown to exert widespread detrimental effects on systemic health with a notable impact on the central nervous system (CNS) 3. Accumulating evidence suggests that CIH triggers a cascade of pathophysiological events, including oxidative stress, neuroinflammation, and neuronal apoptosis, which promote neural injury and a range of neurological dysfunction 4,5. OSA/CIH has been increasingly recognized as a risk factor for the progression of neurodegenerative disorders, such as Parkinson's disease (PD) 6,7. Clinical studies demonstrate that OSA elevates PD risk 8 and accelerates motor dysfunction in OSA patients 9. Elevated plasma α-synuclein (α-syn) levels and reduced dopamine (DA) availability in the caudate nucleus have also been observed in OSA patients 10,11. However, research investigating the effects of OSA/CIH on PD and its underlying mechanisms remains relatively limited.

As the second most common neurodegenerative disorder, PD presents as progressive motor dysfunction and imposes substantial socioeconomic burdens. Its primary pathology involves loss of DA neuron in the substantia nigra pars compacta (SNc) and the aggregation of misfolded α-synuclein 12. According to epidemiological research, OSA affects 45% of PD patients, and it is linked to greater cognitive and motor impairment as well as a higher risk of PD 13. In addition, preclinical investigations indicate that CIH exacerbates oxidative stress, mitochondrial dysfunction, neuroinflammation, and apoptosis in DA neurons 14. Despite this converging clinical and pathological evidence linking OSA/CIH to PD, the mechanistic basis for CIH-induced PD susceptibility remains unresolved.

Here, we investigate the impact of CIH on PD susceptibility and its underlying molecular mechanisms. Using subtoxic PD mouse models, we demonstrate that CIH exacerbates motor deficits and DA neuropathology by inducing pathological LD accumulation. Mechanistically, CIH triggers mitochondrial hyperfission and disrupts LD-mitochondrial interactions in DA neurons, leading to impaired lipid catabolism and aberrant LD deposition. We further identify that peroxisome proliferator-activated receptor α (PPARα)-mediated tethering between mitofusin-2 (Mfn2) on mitochondria and perilipin-5 (Plin5) on LDs is essential for maintaining LD-mitochondrial interactions in DA neurons. Our findings uncover a novel LD-mitochondrial axis as a nexus between CIH and PD, offering a potential targetable pathway for therapeutic intervention in OSA-associated PD risk.

## Materials and Methods

### Animals

Eight-week-old male C57BL mice were employed for experimentation in the current study. All experimental protocols were approved by the Fudan University Animal Care and Use Committee (No. 20240513S) and were conducted following the ARRIVE guidelines for reporting animal research and NIH standards 15. The mice had unlimited access to food and water and were kept in a controlled environment with a 12-h light/dark cycle. Random allocation to experimental groups was performed, and experiments were carried out following standardized laboratory procedures involving randomization and blinding.

### Cell culture

BV2 microglial cells (Corning Costar, MA, USA) and MN9D DA cells (Puhongnuo, Shanghai, China) were cultured in DMEM (Gibco, MA, USA) with 10% fetal bovine serum (FBS) and 1% penicillin-streptomycin. The cells were all incubated at 37 °C with 5% CO_2_. All cell lines used in this study have been authenticated and confirmed free of common misidentifications listed in the cellosaurus database.

For primary midbrain neuron cultures, midbrains were dissected from C57BL/6 mice at E14.5. After careful removal of the meninges, the tissues were minced into small fragments with sterile scissors and digested with 0.0625% trypsin at 37 ℃ for 10 min. The digested tissue was then gently triturated to obtain a single-cell suspension, which was subsequently plated onto plates coated with poly-L-lysine. After 4 h of attachment, the medium was replaced with neurobasal medium (Gibco, USA) supplemented with 2% B27 (Gibco, USA) and 1% GlutaMax (Gibco, USA). For primary microglia cultures, brain tissues were obtained from P1 C57BL/6 mice after the removal of the meninges to generate a single-cell suspension. The cells were cultured in DMDM with 10% FBS. After 14 days in culture, the microglia were dissociated using 0.0625% trypsin for 30 min at 37 ℃ and subsequently plated onto PDL-coated coverslips for further analysis.

### CIH exposure

The CIH protocol was modified based on our previous research 5,16. CIH exposure was implemented using a specialized apparatus (Oxycycler Model A84XOV, Biospherix, Redfield, NY), which includes nitrogen and oxygen tanks, an oxygen sensor, and a circulating fan. Mice spent 8 h a day, from 9:00 AM to 5:00 PM, in a CIH chamber. During the modeling period, the controller injected nitrogen into the chamber to reduce oxygen concentration to 6 ± 1% (maintained for 30 s), followed by oxygen injection to restore oxygen concentration to 21% (maintained for 30 s), completing one intermittent hypoxia cycle. Control mice were housed under normoxic conditions in the same environment. To evaluate the effect of CIH on motor function alone, the exposure duration was set at 10 weeks, which represented the longest period that did not induced significant weight loss and thus avoided confounding effects on motor performance 17,18. In subtoxic PD models, mice were subjected to 4 weeks of CIH exposure to mimic the nocturnal hypoxemia observed in moderate OSA patients and to investigate the specific impact of CIH on PD susceptibility 19.

For the CIH treatment* in vitro*, cells were cultured in a chamber cycling between normoxia (21% O_2_, 30 min) and hypoxia (1% O_2_, 30 min) for 6 h 20. In the CIH+MPP^+^ group, cells were pretreated with a subtoxic dose of MPP^+^ (100 μM) for 6 h of CIH exposure. Corresponding control cells were maintained under normoxic conditions and treated with PBS.

### PD mouse models

To establish a subtoxic MPTP model, mice were administered a subtoxic dose of MPTP (10 mg/kg; i.p.) 21. For the CIH + sMPTP model, after 30 days of CIH pre-treatment, mice received MPTP (10 mg/kg) for 5 consecutive days. In the CIH+aMPTP model, mice received four subtoxic dose injections of MPTP (10 mg/kg) at 2-h intervals after 30 days of CIH pre-treatment. Behavioral tests and sample collection were conducted 3 days after the last MPTP injection.

To establish the CIH+α-syn PFFs model, subtoxic α-syn PFFs were intracranially injected into the bilateral striatum. α-syn PFFs (1 mg/mL, ab246002, Abcam) were sonicated for 10 cycles (30 s on, 30 s off) using a Covaris E200 Focused Ultrasound Device. 1 μL α-syn PFFs (purity > 95%) was bilaterally injected into the striatum (0.21 mm lateral to the midline, 0.07 mm anterior to bregma, and 0.35 mm below the pia) of 8-week-old C57BL male mice with a microinjection pump (NanJect III, Drummond Scientific Company). Control mice were injected with 1 μL PBS. 2 months after injection, mice were subjected to CIH treatment for an additional 4 weeks.

### Drug administration

To activate ATGL, Compound 86 (80 mg/kg; MCE) was delivered via oral gavage daily for a total of 7 days 22. For mitochondrial division inhibition, Mdivi-1 (50 mg/kg, Sigma-Aldrich) was administered intraperitoneally once every day over a 5-day period 23. To activate PPARα, mice were maintained on a diet supplemented with 0.2% fenofibrate (SYSE Inc., Changzhou, China) throughout the experiment period 24.

To locally activate PPARα in the SNc region, we performed cannula implantation and local infusion of GW7647, a PPARα agonist. A custom-made 27-gauge stainless-steel guide cannula was unilaterally implanted targeting the SNc (coordinates: 2.8 mm posterior to bregma, 1.3 mm lateral to the midline, and 4.6 mm below the pia) to allow repeated infusion of GW7647. For drug delivery, a 4-mm guide cannula was paired with an infusion cannula protruding 1 mm beyond its tip. One week after cannula implantation, drug infusion was initiated. GW7647 (5 μg/μL) was dissolved in vehicle (VEH) solution consisting of 2% DMSO, 3% Tween80 and 95% saline and administered twice a week for 4 weeks (0.5 μL infused over 2 min). Control animals received an identical volume of VEH solution.

To activate ATGL, MN9D cells were pretreated with Compound 86 (MCE, 100 nM) for 24 h. For mitochondrial fission inhibition, MN9D cells were pretreated with Mdivi-1 (Sigma-Aldrich, 20 μM) for 2 h. To activate PPARα, MN9D cells were pretreated with fenofibrate (MCE; 100 μM) for 12 h. To competitively inhibit APOE-mediated lipid uptake, low-density lipoprotein (LDL; ThermoFisher Scientific; 5 μg/mL) was added for 6 h. Triglyceride (TG) and cholesteryl ester (CE) assay kits (Nanjing Jiancheng, China) were used to determine TG and CE concentrations in the culture supernatants. In addition, exogenous TG (Aladdin, 0.15 mM) and CE (Aladdin, 0.3 mM) were supplemented to BV2 for 24 h.

### Co-culture of BV2 and MN9D cells

In the media transfer assay, MN9D cells were exposed to CIH and/or MPP^+^, and the medium was resubstituted with serum-free DMEM. After 24 h, the conditioned medium (CM) was obtained. The BV2 cells were exposed to experimental media consisting of 50% CM from the various MN9D treatment groups and 50% fresh complete medium for 24 h.

For the fluorescent fatty acid (FA) pulse-chase experiment, MN9D cells were plated on glass coverslips and labeled by incubating with 1 μM BODIPY™ 558/568 C12 (Red C12, ThermoFisher Scientific) for 16 h. Following labeling, cells were washed to remove excess fluorophore. Then, these labeled MN9D cells on coverslips were transferred to 6-well plates. BV2 cells, seeded on separate coverslips, were placed in the same wells. The co-cultures were then subjected to CIH and/ or MPP^+^ treatments.

### Open field test (OFT)

Briefly, 24 h prior to testing, animals were acclimated in a 42 × 42 × 50 cm chamber. On the experimental day, mice were again placed in the same chamber for 10 min under red light. The chamber floor and walls were thoroughly cleaned between each mouse trial. The total distance traveled within 10 min in the chamber was measured.

### Pole test

Briefly, one day before the experiment, mice underwent adaptive training. Mice were placed at the top of a 55-cm high vertical pole and were allowed to climb down the pole three times with their heads facing up and three times with their heads facing down. On the experiment day, mice were positioned at the top of the pole, facing upwards, and the duration required for them to reach the bottom was recorded.

### Hang test

The hang test apparatus consists of a 23-cm-long horizontal wire 50 cm above the ground, with two platforms on each side. Mice underwent a 1-min habituation period on each platform before the experiment. Then, mice were required to grasp the horizontal wire with their forelimbs, and the numbers of mice climbing onto the platforms and falling off within 3 min were counted. The initial score was set at 10 points. One point was deducted every time mice fell off, and one point was added every time mice reached the platform. The experiment was terminated when a mouse fell off 10 times.

### Rotarod test

In the rotarod test, mice were pre-trained one day before the experiment. Mice were positioned on the rotarod, which was gradually accelerated from 0 to 30 rpm over 4 min. This training protocol was repeated twice with an interval exceeding 1 h. During the experiment, the rotarod was accelerated from 0 to 30 rpm over 90 s, and then maintained at 30 rpm. The time taken for the mice to fall was recorded, with a maximum observation period of 4 min.

### HPLC analysis

Briefly, striatal tissues were dissected, and each sample was treated with 10 μL/mg perchloric acid for tissue precipitation, followed by mechanical homogenization. The homogenates were centrifuged, and the supernatant was collected for analysis using the Agilent 1200 series analyzer (Agilent Technologies, CA, USA). Signal acquisition and data processing were conducted using ChemStation software (Agilent Technologies).

### Lentivirus transfection

To achieve stable overexpression of Mfn2 and Plin5 in MN9D cells, we transduced cells at 30%-50% confluence with Mfn2 or Plin5 lentivirus (Genomeditech, China) at an MOI of 30 along with 8 μg/mL polybrene for 48 h. Then puromycin (3 μg/mL, Invitrogen) was applied for 48 h. Western blotting (WB) was subsequently performed to verify the over-expression levels of Mfn2 and Plin5. MN9D cells transduced with empty vector lentivirus were used as controls.

### WB analysis

Briefly, tissue and cell samples were lysed, sonicated, and centrifugated. Then, the supernatant was collected, denatured and resolved by SDS-PAGE and transferred onto PVDF membranes. After blocking, primary antibodies were added and incubated at 4 °C for 16 h. The membrane was washed and then incubated with the secondary antibodies at room temperature (RT) for 1 h. Protein bands were visualized using enhanced chemiluminescence and quantified. The antibodies used for WB are as follows: rabbit anti-TH (1:3000, AB152, Sigma-Aldrich), rabbit anti-α-syn (1:500, 10842-1-AP, Proteintech), mouse anti-β-actin (1:8000, abs830031ss, Absin), mouse anti-α-tubulin (1:8000, 66031-1-Ig, Proteintech), rabbit anti-GFAP (1:3000, 16825-1-AP, Proteintech), rabbit anti-ACC1 (1:3000, 21923-1-AP, Proteintech), rabbit anti-ATGL (1:1000, 55190-1-AP, Proteintech), rabbit anti-Drp1 (1:2000, 12957-1-AP, Proteintech), rabbit anti-phosphorylated Drp1 (1:1000, 4494S, CST), rabbit anti-Mfn1 (1:5000, 13798-1-AP, Proteintech), mouse anti-Mfn2 (1:5000, 67487-1-Ig, Proteintech), rabbit anti-Plin5 (1:2000, 26951-1-AP, Proteintech), rabbit anti-Plin2 (1:1000, MCE), rabbit anti-PPARα(1:1000, MCE), and mouse anti-LAMP2(1:3000, 66301-1-Ig, Proteintech).

### Immunofluorescence staining

After being fixed in 4% paraformaldehyde (PFA) for 4-6 h, mouse brains were subjected to a sucrose gradient for cryoprotection and embedded in OCT compound. Coronal sections (30 µm thickness) were prepared using a cryotome (Leica CM1950). The sections were permeabilized, blocked, and then incubated with primary antibodies overnight at 4 °C. Cell sample were fixed for 15 min, then permeabilized and blocked. The next day, the sections were incubated with appropriate secondary antibodies at RT for 2 h. For BODIPY staining, the sections were incubated with BD493 (2 μg/μL, D3922, ThermoFisher Scientific) for 30 min at RT. Imaging and photography were performed using a Nikon fluorescence microscope (Nikon-1A, Japan) with a 60× objective. For 3D reconstruction, confocal stacks at 0.5 μm intervals were obtained and analyzed using Imaris software and ImageJ software. The antibodies used were as follows: rabbit anti-TH (1:1000, AB152, Merck), mouse anti-TH (1:1000, ab129991, Abcam), rabbit anti-GFAP (1:1000, 16825-1-AP, Proteintech), goat anti-iba1 (1:100, 011-27991, Wako), rabbit anti-GABA (2.5 μg/mL, A2052, Sigma-Aldrich), rabbit anti-ATGL (1:200, 55190-1-AP, Proteintech), mouse anti-Mfn2 (1:200, 67487-1-Ig, Proteintech), mouse anti-LAMP2 (1:500, 66301-1-Ig, Proteintech), rabbit anti-Plin5 (1:200, 26951-1-AP, Proteintech) and rabbit anti-TOMM20 (1:200, AF1717, Beyotime).

### Image processing and analysis

Three-dimensional reconstruction and volumetric analysis of LDs were performed using Imaris software (v9.5.0, Bitplane AG, Switzerland). Briefly, a mask was created from segmented cellular signals, and then LDs contained in the mask were reconstructed, and volumes were calculated. The relative volume of LDs was calculated as the volume of LDs contained within cells relative to the total volume of cells.

To quantify the level of transferred lipids within BV2 cells, images were subjected to Imaris software. BD493-positive LD signals and BD558/568 C12-positive signals were reconstructed, and volumes were calculated separately. The volume of Red-C12 positive puncta versus total BODIPY 493-positive LD volume was calculated and analyzed.

For mitochondrial morphology analyses, the ImageJ software was utilized to calculate the mitochondrial aspect ratio (AR, major/minor axis of an ellipse)) and the form factor (FF, perimeter^2^/4π∙area) 25. Decreases in AR and FF values indicate mitochondrial fragmentation.

For colocalization analyses, a Spin FV-COMB confocal spinning disk microscope was employed (60× objective lens, 3× digital zoom, 0.5 μm intervals along the Z-axis). The Mander's coefficient was calculated using the ImageJ plugin “JACoP” 26.

### Quantification of SNc TH+ cells

TH^+^ cells in SNc were counted using unbiased stereological methods as previously described 27. For each mouse, we systematically sampled one out of every two 40-μm-thick coronal sections spanning the SNc region (bregma -2.80 to -3.64 mm anteroposterior coordinates), yielding a total of 8 representative sections per animal for immunohistochemical processing and quantitative analysis. All sections were imaged using the Olympus Virtual Slide Scan System (VS120) equipped with a 20× objective lens, acquiring three z-stack images at 5 μm intervals. The SNc region was delineated based on Paxinos mouse brain atlas, and the number of SNc TH^+^ neurons was stereologically counted.

### Transmission electron microscopy (TEM)

MN9D cells were harvested and fixed in 2.5% glutaraldehyde at RT for 30 min, then stored at 4 °C. Subsequently, the samples were dehydrated using an ethanol gradient and infiltrated with 100% propylene oxide. After infiltration, cells were embedded in epoxy resin and polymerized for 24 h. A Leica UC7 ultramicrotome was used to create 70 nm thick ultrathin sections. Uranyl acetate and lead citrate were then used for staining. Finally, imaged were obtained using Hitachi HT7800 transmission electron microscope.

### Co-immunoprecipitation (Co-IP)

Tissue and cell samples were lysed using IP lysis buffer supplemented with PMSF. For tissues, samples were homogenized using a mechanical homogenizer (Tisselyser-24, Shanghai Jingxin). The lysates centrifuged and the supernatant was divided into three portions: one was incubated with the primary antibody (IP group), one with IgG (IgG group), and the remaining portion was directly stored at -80 °C (Input group). The IP and IgG groups were incubated and rotated overnight at 4 °C. On the following day, protein A+G beads (40 μL) were added and incubated on the rotator at 4 °C for 6 h. After washing, protein complexes were eluted. Eluates from IP, IgG, and input controls were resolved by SDS-PAGE and subjected to immunoblotting using anti-Mfn2 (1:5000, 67487-1-Ig, Proteintech) and anti-Plin5 (1:2000, 26951-1-AP, Proteintech) antibodies.

### Proximity Ligation Assay (PLA)

The PLA experiments were carried out using Duolink PLA kit (DUO92101, Sigma-Aldrich). Cells were fixed, permeabilized, and blocked as before mentioned. Cells were incubated overnight at 4 °C with primary antibodies: mouse anti-Mfn2 (1:200; Proteintech, 67487-1-Ig) and rabbit anti-Plin5 (1:200; Proteintech, 26951-1-AP). On the next day, oligonucleotide-conjugated secondary antibodies were applied. Following ligation and rolling-circle amplification, the amplicon was hybridized with labeled oligonucleotides to visualize the PLA signals.

### Transcriptomic analyses

RNA sequencing was performed to compare transcriptomic profiles in SN between NOR+sMPTP and CIH+sMPTP treatment groups. Total RNA was isolated, followed by poly(A)+ mRNA enrichment using the TIANGEN TIANSeq mRNA Capture Kit (TIANGEN Biotech, Beijing). Sequencing libraries were prepared, and high-throughput sequencing was conducted on an Illumina NovaSeq 6000 platform. Genes with |log₂ fold change| > 0.26 (approximately 1.2-fold) and an FDR-adjusted* P* < 0.05 were considered differentially expressed genes (DEGs). DEGs were subjected to enrichment analyses using GO and KEGG databases, implemented via the cluster Profiler package (v4.0.5) in R (v4.1.2). Gene lists associated with mitochondrial dynamics were curated from the MitoCarta 3.0 database 28. Lists of lipid metabolism-related genes were obtained from Gene Ontology (GO) terms (GO:0006631, GO:0006638, GO:0016127, GO:0010867, GO:0010868, and GO:0045923) 29. Three distinct gene sets were investigated: 1) total DEGs, 2) lipid metabolism-associated DEGs, and 3) DEGs involved in lipid metabolism and mitochondrial dynamics.

### Untargeted lipidomics analysis

Tissue samples were homogenized in the extraction buffer (50 Hz, 60 s, 2 cycles). Then, the lysates were sonicated and centrifuged to collect supernatants. The samples were vacuum-dried, and reconstituted in solvent for LC-MS analysis. Raw data were processed using ProteoWizard (v3.0.20) and the R (based on XCMS algorithms) for feature detection, peak alignment, and integration. Metabolite annotation was performed by matching MS/MS spectra against an in-house lipid database with a dot product cutoff score ≥ 0.3. Principal component analysis (PCA) and partial least squares-discriminant analysis (PLS-DA), were conducted to identify intergroup differences. Hierarchical clustering analysis was implemented in R, and differential metabolites were further validated using GraphPad Prism (v9.5).

### Statistical analysis

Statistical analyses were conducted using GraphPad Prism 9.5 software, and data were expressed as the mean ± standard error of the mean (SEM). The Shapiro-Wilk test was used to evaluate normality. For normally distributed data, two-tailed Student's t-test, one-way ANOVA followed by Bonferroni post hoc test, or two-way ANOVA followed by Tukey's post hoc test were utilized. The Mann-Whitney or Kruskal-Wallis tests were applied for data that did not follow a normal distribution. A two-tailed *P* < 0.05 defined statistical significance. The figure legends include information about statistical analyses and the number of individual mice or experiments (n).

## Results

### CIH exacerbates nigrostriatal DA degeneration and motor deficits in subtoxic PD models

**Figure 1:**
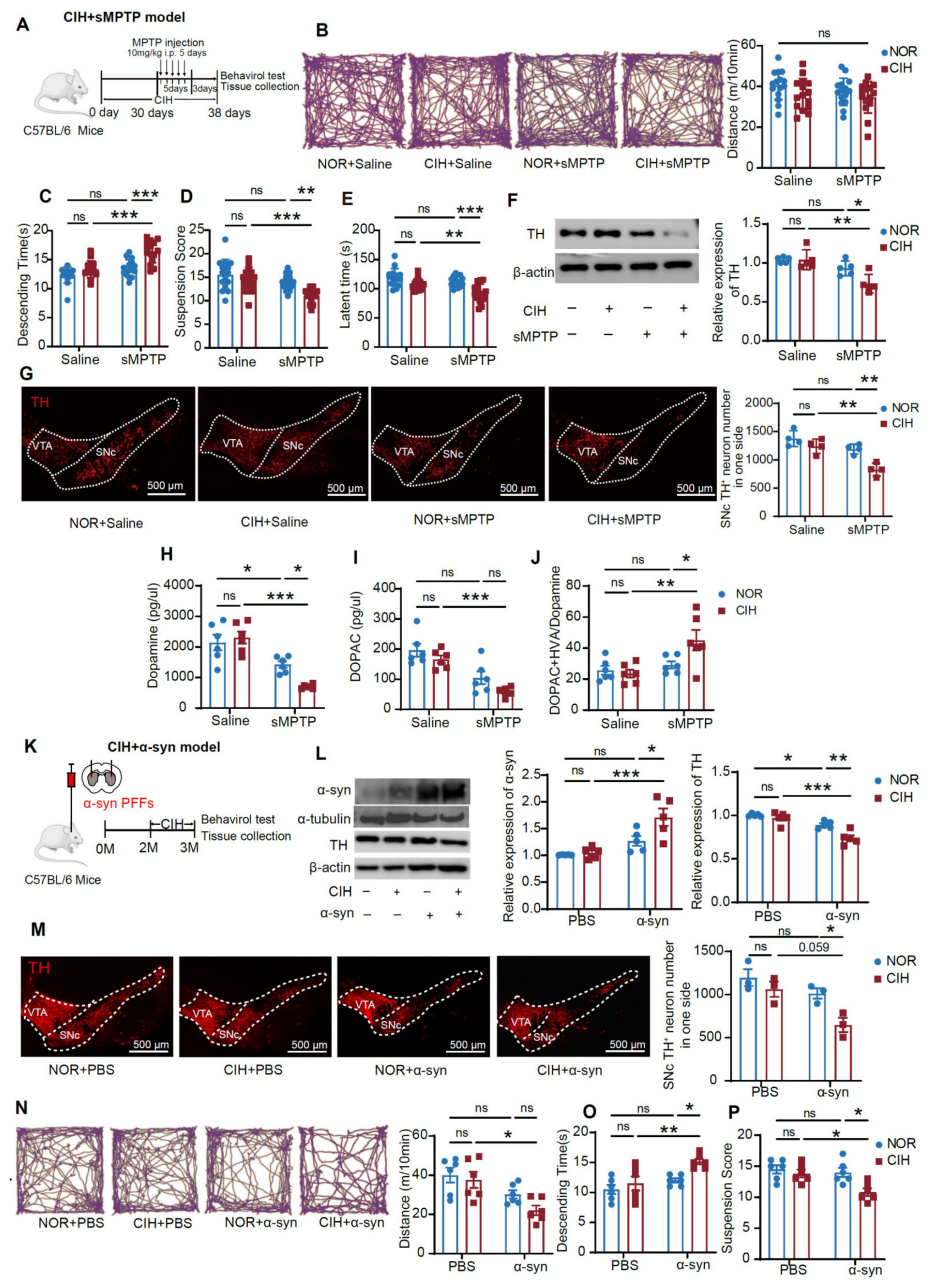
CIH exposure exacerbates nigrostriatal DA degeneration and motor deficits in subtoxic PD models.** (A)** Schematic diagram of CIH+sMPTP exposure protocol. **(B-E)** Example trajectories and total distance traveled in the OFT (b), descending time in the pole test (**C**), suspension score in the hang test (**D**), and time spent on the rotarod (**E**). n = 15 mice in each group. (**F**) Examples and summary of striatal TH expression. n = 5 mice in each group.** (G)** Representative images and statistical numbers of TH^+^ cells in one SNc side, n = 4 mice per group. **(H-J)** HPLC analyses of dopamine (**H**), DOPAC (**I**), and the ratio between DOPAC+HVA and DA (**J**) in the striatum. n = 6 mice in each group. **(K)** Schematic diagram of CIH+α-syn exposure protocol. **(L)** Expression levels of striatal α-syn and TH. n = 5 mice in each group. **(M)** Representative images and statistical analysis of TH^+^ neurons in one SNc side. n = 3 mice per group. **(N)** Example trajectories and total distance traveled among different groups in the OFT. n = 6 mice in different groups. **(O-P)** Descending time (**O**) and Suspension score (**P**) in pole test and hang test. n = 6 mice in different groups. Two-way ANOVA with Tukey's post hoc multiple comparisons test for all comparisons. ns = no significant, * *p*<0.05, ** *p*<0.01, *** *p*<0.001.

CIH exposure for around 4 weeks is commonly employed to stimulate the nocturnal fluctuations observed in moderate OSA patient, and exposure durations of up to 10 weeks are used in mice to avoid the confounding effects of body weight changes 30. We observed that a 10-week exposure to CIH alone did not significantly alter mouse motor functions, as demonstrated by the OFT, hang test, and pole test (**Figure S1A-D**). However, a 30-day CIH treatment significantly aggravated motor deficits in the subacute subtoxic MPTP model (CIH+sMPTP), as evidenced by longer descending time in the pole test, lower scores in the hang test, and less time spent on the rotarod, although there was no significant difference in total travel distance in the OFT (**Figure 1A-E**). Neuropathological analyses revealed that CIH+sMPTP treatment significantly reduced striatal tyrosine hydroxylase (TH) expression and the number of TH^+^ neurons in SNc, whereas subtoxic MPTP or CIH alone had no effects (**Figure 1F-G**). Given that SNc DA neurons target the striatum to release DA and modulate motor functions, we evaluated changes in striatal DA levels and its downstream metabolites. Compared with the subtoxic MPTP or CIH alone groups, the CIH+sMPTP treatment decreased DA and 3,4-dihydroxyphenylacetic acid (DOPAC) levels and increased the ratio between DOPAC+homovanillic acid (HVA) and DA (**Figure 1H-J**).

We confirmed the influence of CIH exposure on PD susceptibility in other subtoxic PD models. In the acute subtoxic MPTP (aMPTP) model, a 30-day CIH pre-treatment significantly exacerbated motor deficits and nigrostriatal DA degeneration (**Figure S1E-J**). In the α-synuclein preformed fibrils (α-syn)-induced subtoxic PD model, CIH exposure elevated the accumulation of α-syn protein (**Figure 1K-L**). Compared to mice treated with CIH or α-syn alone, CIH+α-syn-treated mice exhibited a significant reduction in striatal TH protein levels and SNc TH^+^ neurons (**Figure 1L-M**). Motor deficits were also observed in the CIH+α-syn group (**Figure 1N-P**). These results suggest that, although CIH exposure alone does not affect mouse motor activity, it increases the vulnerability of nigrostriatal DA pathways and exacerbates motor dysfunctions in different subtoxic PD models.

### CIH impairs lipid droplet (LD) homeostasis in subtoxic PD mouse models

**Figure 2:**
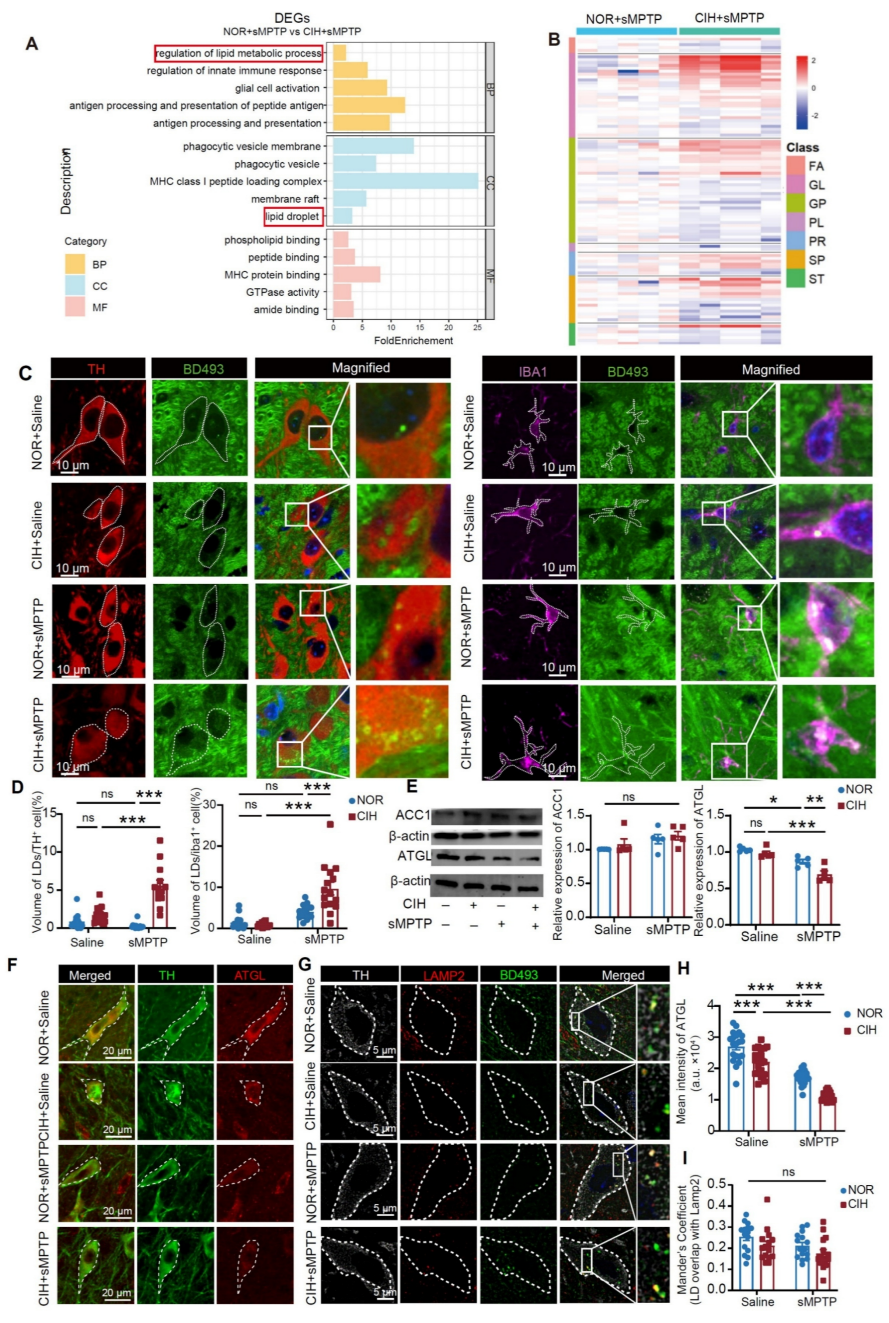
CIH impairs lipid homeostasis in subtoxic PD mouse models**. (A)** GO functional enrichment analysis of DEGs between NOR+sMPTP and CIH+sMPTP groups. n = 3 mice per group. **(B)** Heatmap visualization of SN lipid levels in NOR+sMPTP and CIH+sMPTP groups. n = 5 mice in different groups. Lipid species are identified by rows, while samples are represented by columns. **(C)** Representative images of TH, Iba1, and LD immunofluorescence staining in SNc. **(D)** Statistical analysis of LD volume in TH^+^ and Iba1^+^ neurons. n = 15 cells randomly selected from 3 mice. **(E)** Expression levels of ACC1 and ATGL in SN. n = 5 mice in different groups. **(F-G)** Representative immunofluorescence images of TH and ATGL (**F**) and LAMP2 and LD (**G**) staining in SNc. **(H-I)** Statistical analysis of mean intensity of ATGL in TH^+^ cells (**H**) and quantification of LAMP2-LD colocalization by Mander's coefficient (**I**). n = 15 cells randomly selected from 3 mice. Two-way ANOVA with Tukey's post hoc multiple comparisons test for all comparisons. ns = no significant, * *p*<0.05, ** *p*<0.01, *** *p*<0.001.

To investigate how CIH enhances PD susceptibility in subtoxic PD models, we performed RNA-sequencing on SN regions from NOR+sMPTP and CIH+sMPTP groups. DEGs were significantly enriched in “regulation of lipid metabolic process” (GO:0019216) and "lipid droplet (LD)" (GO:0005811) (**Figure 2A** and **Figure S2A-B**), suggesting the possible contribution of compromised lipid metabolism to the CIH-induced PD vulnerability. Untargeted lipidomic analysis revealed obvious lipid profile alterations between NOR+sMPTP and CIH+sMPTP groups (**Figure 2B** and **Figure S2C-D**), especially elevated levels of triglycerides, the major LD components, in the CIH+sMPTP group (**Figure S2E**).

Given established links between pathological LD accumulation and neurodegeneration 31, we hypothesized that CIH exposure disrupted LD homeostasis to promote nigrostriatal DA degeneration in subtoxic PD models. Immunofluorescence staining revealed significantly higher levels of LDs accumulated in SNc DA neurons and microglia of CIH+sMPTP mice compared to other groups (**Figure 2C-D**). Similar results were observed in the CIH+α-syn treated mice (**Figure S3A**), while no significant changes in LD levels were detected in SNc astrocytes and GABAergic neurons (**Figure S3B**). LD accumulation may arise from dysregulated lipid homeostasis, involving lipid biosynthesis and LD degradation through lipolysis and lipophagy pathways 32. While the expression of acetyl-CoA carboxylase 1 (ACC1), a rate-limiting enzyme in lipid synthesis, remained unchanged across different groups, the expression of adipose triglyceride lipase (ATGL), a rate-limiting lipolytic enzyme, was significantly reduced in the CIH+sMPTP group (**Figure 2E**). Similarly, ATGL expression was also significantly reduced in the CIH+α-syn group, while ACC1 expression showed no significant change (**Figure S3C**). We further investigated the changes in lipolysis and lipophagy levels in SNc DA neurons by detecting ATGL levels and LD levels within lysosomes. The results indicated a significantly decreased expression of ATGL in SNc DA neurons of the CIH+sMPTP group (**Figure 2F, H**), while no substantial alterations in LD levels within lysosomes (**Figure 2F-I**). Collectively, these results indicate that CIH impairs lipolysis-mediated LD degradation in DA neurons and disrupts lipid homeostasis in PD-susceptible mice.

### The transfer of lipids from DA neurons to microglia is mediated by APOE and exacerbates neuroinflammation

**Figure 3:**
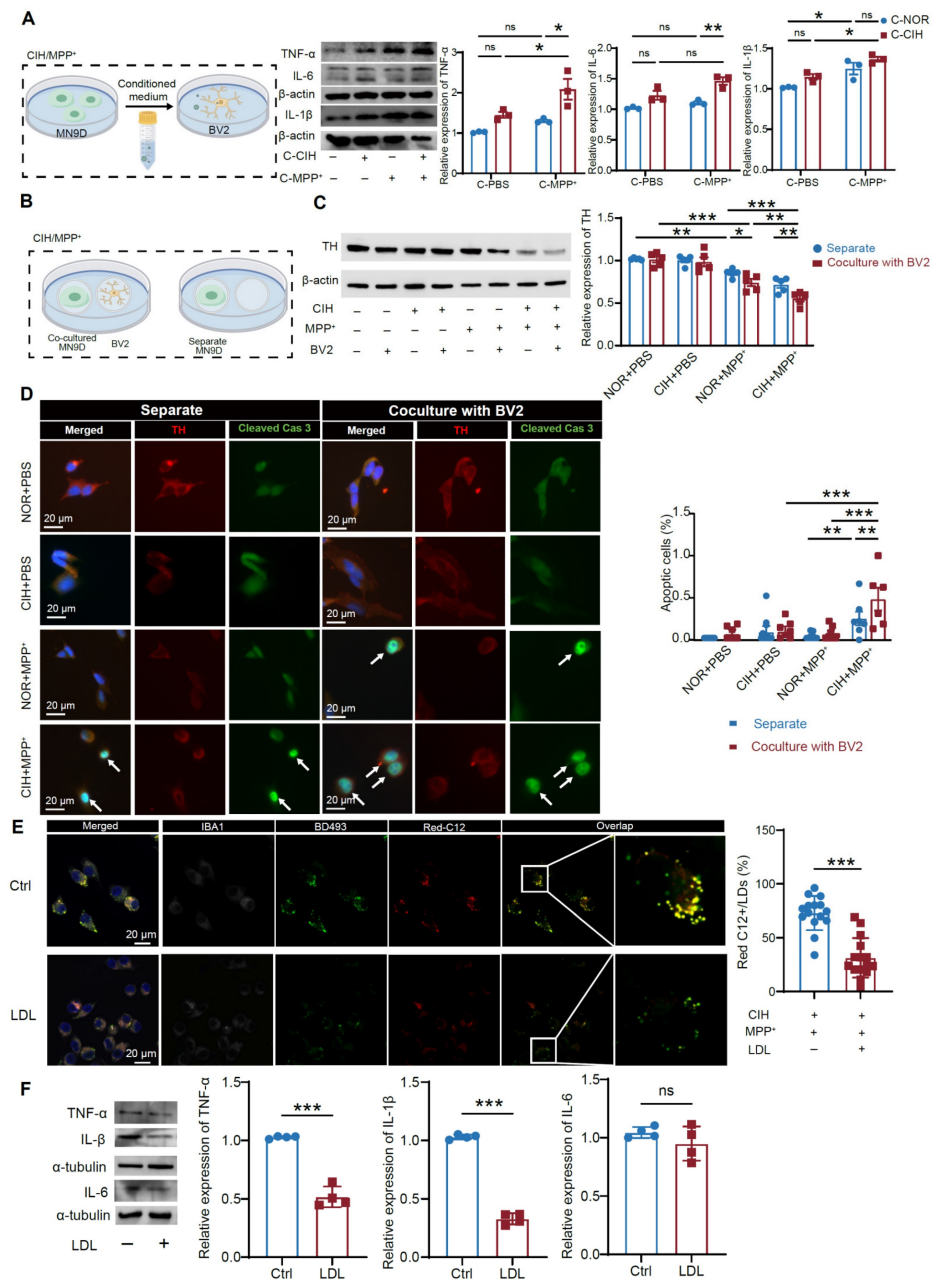
APOE-mediated LD transfer from DA neurons to microglia exacerbates neuroinflammation and DA neuropathology induced by CIH. **(A)** Schematic representation of the indirect coculture of MN9D and BV2 cells and expression levels of TNF-α, IL-6, and IL-1β. n = 3 independent experiments. **(B)** Schematic representation of the separate and co-culture of MN9D and BV2 cells. **(C)** TH expression levels in different conditions. n = 5 independent experiments. **(D)** Representative images of immunofluorescence staining for cleaved caspase-3 in MN9D and statistical analysis of the proportion of apoptotic cells. n = 6 fields randomly selected from 3 independent experiments. **(E)** Representative immunofluorescence images o BD493, Red-C12, and Iba1 staining in BV2 and statistical analysis of the proportion of Red C12/BD493. n = 15 cells randomly selected from 3 independent experiments. **(F)** TNF-α, IL-1β, and IL6 expression levels in different conditions. n = 4 independent experiments. Two-tailed Student's t-test was used for (**E, F**). Two-way ANOVA following with Tukey's post hoc multiple comparisons test for other comparisons. ns = no significant, * *p*<0.05, ** *p*<0.01, *** *p*<0.001.

Molecules secreted by DA neurons, such as cytokines and neurotransmitters, have been implicated in modulating microglial function, initiating vicious cycles of neuroinflammation and neurodegeneration 33. Intracellular lipids within tauopathic neurons have been found to be transferred to microglia, contributing to neuroinflammation and tau pathology 29. We speculated that lipids accumulated in DA neurons may mediate microglial lipid acquisition to modulate microglial functional states. Consistent with findings in animal models, CIH and subtoxic MPP^+^-treated (CIH+MPP^+^) elicited pronounced LD accumulation in murine MN9D dopaminergic neuronal cell lines as well as in primary midbrain neurons of mice (**Figure S4A**). Moreover, CIH+MPP^+^ treatment did not significantly alter LD biogenesis or lipophagy but was associated with a pronounced suppression of lipolysis *in vitro* (**Figure S4B-C**). We then conducted a Red-C12 pulse-chase assay in MN9D-BV2 cocultures and observed elevated intercellular lipid transfer in the CIH+MPP^+^ group (**Figure S5A-B**). Besides, using CM from differentially treated MN9D cells to culture BV2 microglial cells (**Figure S5C**), we observed that CM derived from CIH+MPP^+^-treated MN9D cells induced significant LD accumulation in BV2 microglia (**Figure S5D**). Similarly, CM from CIH+MPP^+^-treated primary midbrain neurons induced substantial LD accumulation in primary microglia (**Figure S5E**). Notably, microglia with lipid accumulation exhibited a pro-inflammatory phenotype characterized by elevated secretion of pro-inflammatory cytokines (**Figure 3A**), which further exacerbated DA neuronal pathology, as evidenced by reduced TH expression and increased levels of cleaved caspase-3 in co-cultured MN9D cells (**Figure 3B-D**). These findings suggest a pathogenic cascade wherein CIH-induced DA neuronal lipid dysregulation promotes microglial lipid accumulation and neuroinflammation, which worsens DA neurodegeneration.

Previous studies have established apolipoprotein E (APOE)-dependent lipid transfer as a classical mechanism of intercellular lipid transfer in the CNS 34. We therefore tested whether APOE mediates the CIH+MPP^+^-induced lipid transfer from DA neurons to microglia. In the neuron-microglia coculture system, we supplemented excess low-density lipoprotein (LDL) to competitively bind LDLR, thereby blocking microglial uptake of other lipids (**Figure S6A**). Quantitative analysis revealed that LDL treatment significantly reduced neuronal Red-C12 lipid transfer into BV2 cells (**Figure 3E**) and attenuated the expression of pro-inflammatory cytokines in cocultured microglia (**Figure 3F**), indicating that APOE is required for DA neuron-microglia lipid crosstalk and its pro-inflammatory effects. In addition, we found that TGs and CEs—the major constituents of LDs—were markedly elevated in the supernatant of CIH+MPP^+^-treated MN9D neurons (**Figure S6B-C**). Supplementation of TG or CE was sufficient to induce pro-inflammatory activation in BV2 cells (**Figure S6D-E**). Taken together, these findings demonstrate that APOE-mediated transfer of TG and CE represents a key mechanism driving DA neuron-microglia lipid crosstalk, which promotes LD accumulation and inflammatory phenotypes in microglia.

### LD-mitochondrial interaction mediates CIH-induced PD susceptibility in MPTP-related mouse and cell models

**Figure 4:**
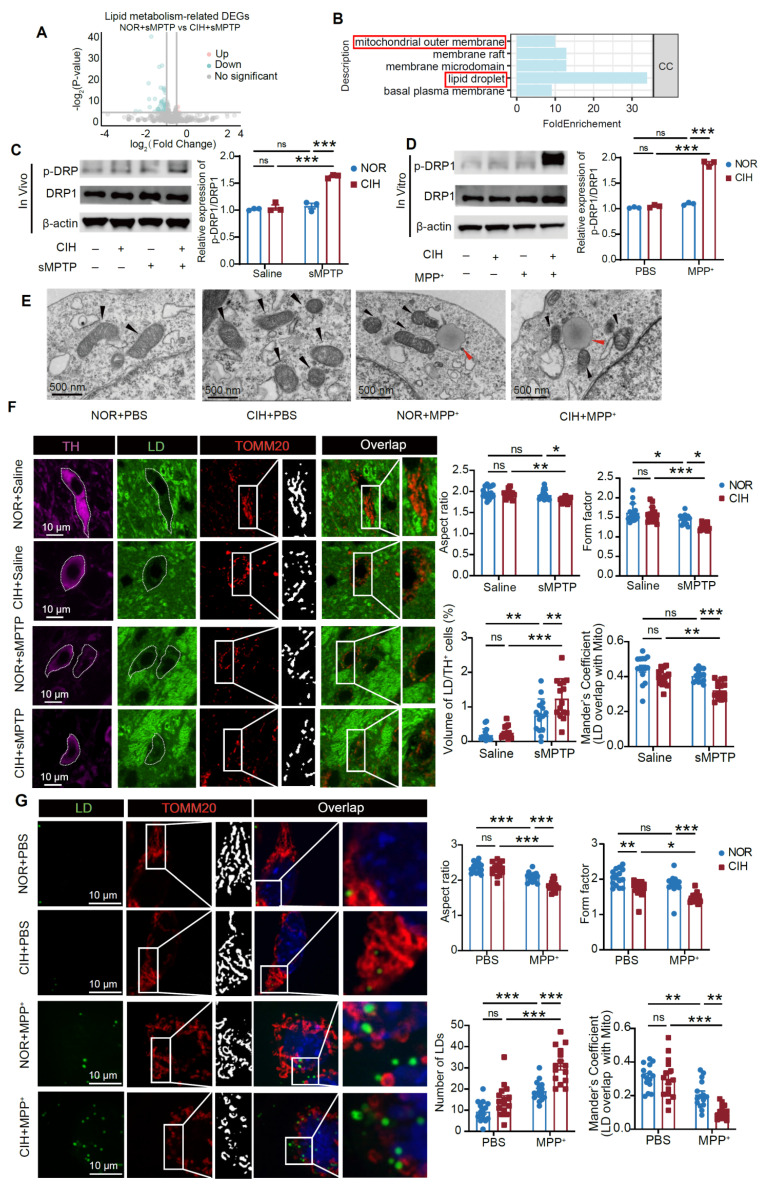
CIH induces mitochondrial fragmentation and impairs LD-mitochondrial interaction in DA neurons of subtoxic PD mouse and cell models. **(A-B)** Volcano plot (**A**) and GO functional enrichment analysis (**B**) of lipid metabolism-related DEGs between NOR+sMPTP and CIH+sMPTP-treated mice. n = 3 mice in each group. **(C)** Levels of p-Drp1/Drp1 in SN in different groups. n = 5 mice among different groups. **(D)** Levels of p-Drp1/Drp1 in MN9D cells in different conditions. n = 3 independent experiments. **(E)** TEM images of MN9D across different groups. The red arrows highlight LDs, and the black arrows highlight mitochondria. **(F)** Representative images of TH, LD, and TOMM20 immunofluorescence staining in SNc and statistical analyses of aspect ratio, form factor, volume of LD in TH^+^ cells, and overlap coefficient between LD and TOMM20. n = 15 cells from 3 mice. **(G)** Representative immunofluorescence images of TH, LD, and TOMM20 staining in MN9D cells and statistical analyses of aspect ratio, form factor, volume of LD in MN9D cells, and overlap coefficient between LD and TOMM20. n = 15 cells from 3 independent experiments. Two-way ANOVA with Tukey's post hoc multiple comparisons test for all comparisons. ns = no significant, * *p*<0.05, ** *p*<0.01, *** *p*<0.001.

To elucidate the mechanism underlying lipid metabolism dysregulation under the CIH condition in subtoxic PD models, we analyzed DEGs associated with lipid metabolism pathways (**Figure 4A**). Notably, lipid metabolism-related DEGs between NOR+sMPTP and CIH+sMPTP groups showed significant enrichment in the "mitochondrial outer membrane” component (GO:0005741) (**Figure 4B**), highlighting the potential association of mitochondria in LD metabolism. Given that mitochondrial dynamics have been associated with lipid metabolism 35, we evaluated mitochondrial morphology in DA neurons. CIH+sMPTP treatment markedly induced mitochondrial fragmentation in DA neurons, as demonstrated by increased phosphorylation of Drp1-Ser616 (**Figure 4C-D**) and fragmented mitochondria visualized by TEM (**Figure 4E**). Additionally, immunofluorescence analysis revealed that DA neurons from CIH+sMPTP -treated mice exhibited significantly reduced mitochondrial AR and FF (**Figure 4F**). These morphological alterations provided evidence of mitochondrial fragmentation following CIH+sMPTP treatment. Concurrently, a notable reduction in LD-mitochondrial colocalization and an elevation in LD levels were also observed in DA neurons of CIH+sMPTP-treated mice (**Figure 4F**). In CIH+MPP^+^-treated MN9D cells and primary midbrain neurons, we also observed increased LD accumulation, enhanced mitochondrial fission, and reduced LD-mitochondrial colocalization (**Figure 4G, Figure S7**). These findings indicate that CIH promotes mitochondrial fission and disrupts the LD-mitochondrial interaction in DA neurons in subtoxic PD models.

**Figure 5:**
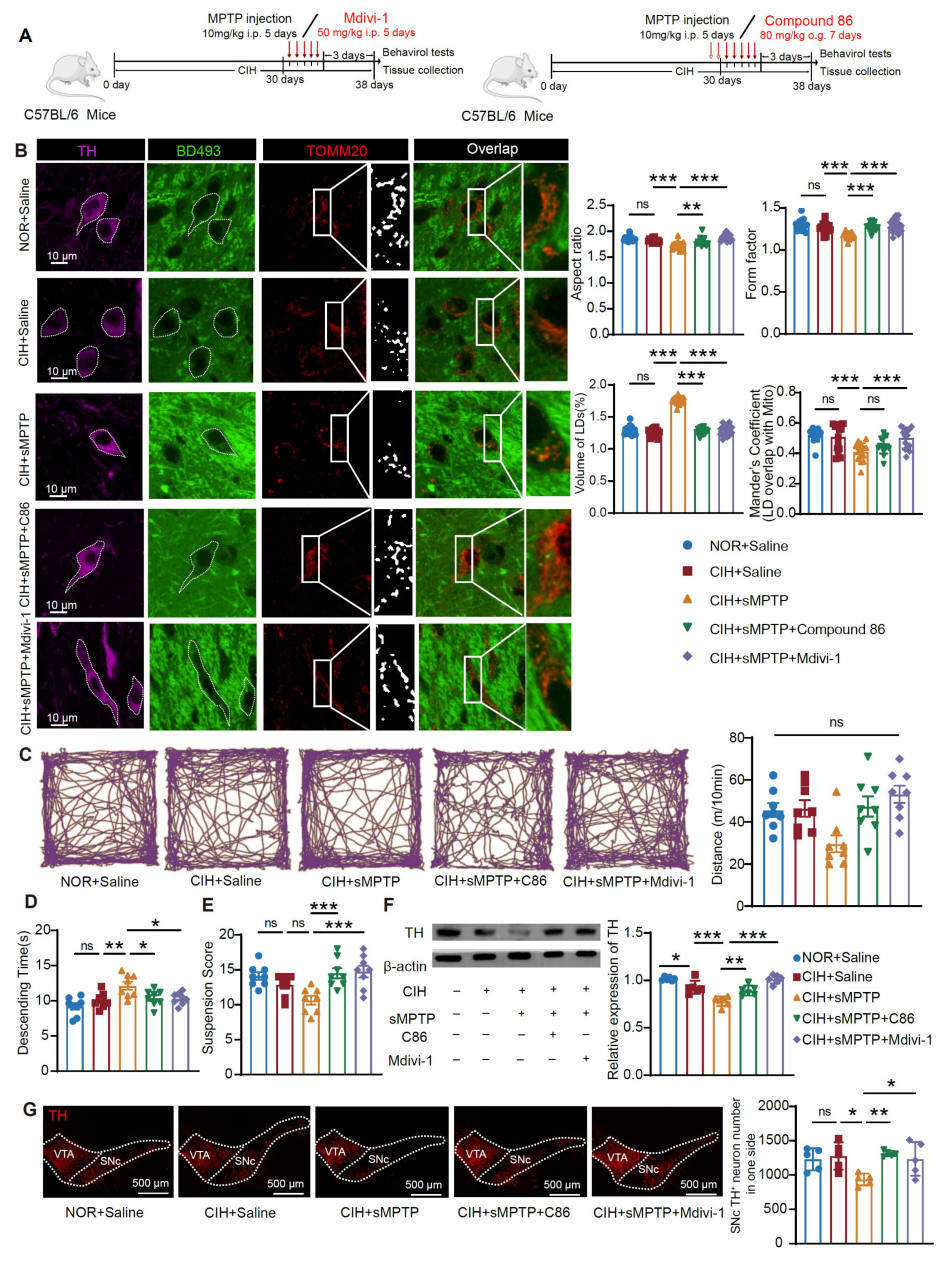
Pharmacological modulation of LD catabolism and mitochondrial fission alleviates the CIH-induced PD susceptibility. **(A)** Schematic diagram of compound 86 and mdivi-1 administration protocols, respectively. **(B)** Left: Representative images of TH, LD, and TOMM20 immunofluorescence staining in SNc in different groups. Right: Statistical analysis of aspect ratio, form factor, volume of LD in TH^+^ cells, and Mander's coefficient between LD and TOMM20. n = 15 cells from 3 mice. **(C)** Example trajectories and total distance traveled in the OFT among different groups. n = 8 mice. **(D-E)** Descending time (**D**) and suspension score (**E**) among different groups. n = 8 mice. **(F)** Expression of TH in the striatum. n = 6 mice for each group. **(G)** Representative images and statistical analysis of TH^+^ cells in one SNc side, n = 5 mice. One-way ANOVA with Bonferroni multiple comparisons test for all comparisons. ns = no significant, * *p*<0.05, ** *p*<0.01, *** *p*<0.001.

To further confirm the roles of LDs and mitochondria in CIH-enhanced PD susceptibility, we employed compound 86 (an ATGL activator) to enhance LD catabolism and mdivi-1 (Drp1 phosphorylation inhibitor) to suppress mitochondrial fission, respectively (**Figure 5A**) 22,23. In the CIH+sMPTP mouse model and CIH+MPP^+^ cultured cells, compound 86 upregulated ATGL expression, and mdivi-1 effectively inhibited Drp1 phosphorylation (**Figure S8A-B**). Immunofluorescence staining demonstrated that both compound 86 and mdivi-1 reduced LD accumulation, restored mitochondrial morphology, and promoted LD-mitochondrial interaction (**Figure 5B**). Consistent findings were also observed *in vitro* (**Figure S8C**). We further evaluated the roles of LD and mitochondrial regulation in motor dysfunction and DA neurodegeneration. Motor performance in OFT, hanging, and pole tests were improved when LD accumulation and mitochondrial fission were suppressed (**Figure 5C-E**), suggesting rescue of PD-related motor deficits. Consistently, both compound 86 and mdivi-1 increased striatal TH expression and preserved SNc TH^+^ neurons in the CIH+sMPTP model (**Figure 5F-G**). Together, these findings demonstrate that CIH-induced PD susceptibility mechanistically depends on disrupted LD metabolism, pathological mitochondrial fission, and impaired LD-mitochondrial crosstalk.

### Mfn2/Plin5 complex mediates LD-mitochondrial contacts in DA neurons

**Figure 6:**
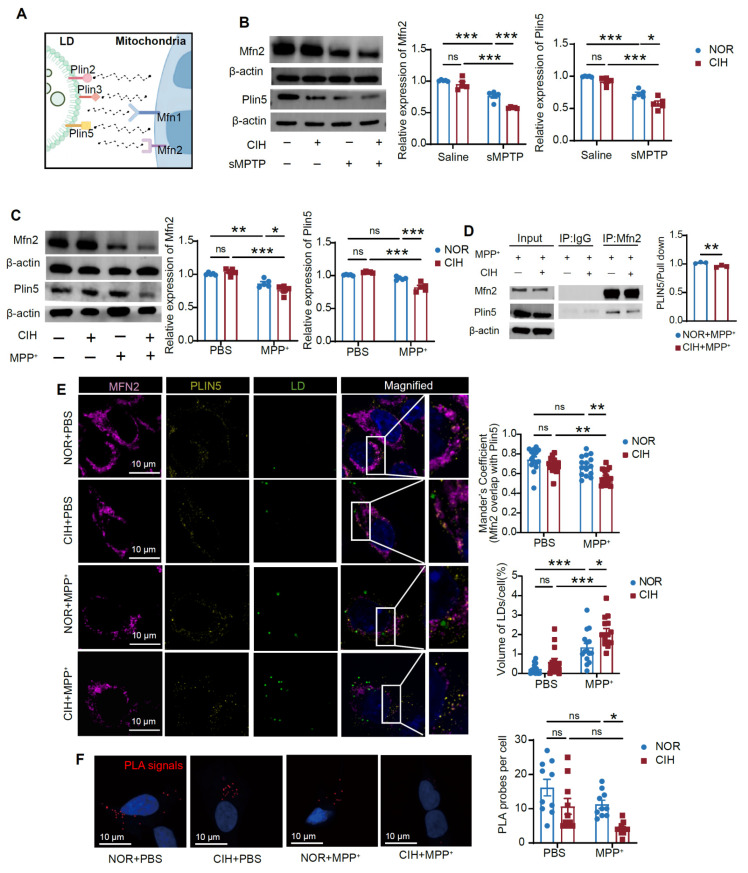
Mfn2/Plin5 complex mediates LD-mitochondrial contacts in DA neurons. **(A)** Hypothetical illustration of the possible protein tethers mediating the interaction between LD and mitochondria. **(B)** Expression of Mfn2 and Plin5 in SN in different conditions. n = 5 mice. **(C)** Expression of Mfn2 and Plin5 in MN9D cells in different conditions. n = 5 independent experiments. **(D)** Co-IP assay using Mfn2 antibody to determine the binding of Mfn2 and Plin5 and statistical analysis of fold change of plin5 pulled down in MN9D cells. n = 3 independent experiments. **(E)** Representative immunofluorescence images of Mfn2, Plin5, and LD staining in MN9D cells and statistical analysis of the Mander's coefficient between LDs and mitochondria and relative volume of LDs. n = 15 cells from 3 independent experiments randomly selected. **(F)** Representative images and quantitative analysis of PLA signals in MN9D cells. PLA signals are visible as red dots. n = 10 cells from 3 independent experiments randomly selected. Two-tailed Student's t-test was applied for Co-IP analysis. Two-way ANOVA with Tukey's post hoc multiple comparisons test was used for other experiments. ns = no significant, * *p* < 0.05, ** *p* < 0.01, *** *p* < 0.001.

Given the role of LD-mitochondrial interaction in CIH-induced PD susceptibility, we further explored the molecular basis of impaired LD-mitochondrial crosstalk. Mitofusin-1/2 (Mfn1/Mfn2), outer mitochondrial membrane proteins regulating mitochondrial fusion, mediate membrane contact site formation with organelles such as the endoplasmic reticulum 36. Perilipins (Plin), the most abundant LD surface proteins, feature brain-expressed isoforms Plin2, Plin3 (glial-specific), and Plin5 37. We thus evaluated the role of Mfn1/Mfn2 and Plin2/Plin5 in mediating impaired LD-mitochondrial contacts (**Figure 6A**). Mfn2 and Plin5 expression levels were significantly downregulated in CIH+sMPTP group (**Figure 6B**), while Plin2 expression was upregulated and Mfn1 expression remained unchanged (**Figure S9A**). Similarly, MN9D cells showed reduced Mfn2 and Plin5 expression, increased Plin2 expression, and unaltered Mfn1 levels in CIH+MPP^+^-treated group (**Figure 6C; Figure S9B**). Co-IP assays demonstrated a direct interaction between Mfn2 and Plin5. This interaction was detected when immunoprecipitating Plin5 with an Mfn2 antibody and reciprocally when immunoprecipitating Mfn2 with a Plin5 antibody. Notably, the Mfn2-Plin5 interaction was significantly reduced following CIH+MPP^+^ treatment (**Figure 6D; Figure S9C**). Mfn2-Plin2 binding was also detected, but this interaction was increased after CIH+MPP^+^ treatment (**Figure S9D**), and no interaction was observed between Mfn1 and Plin5 (**Figure S9E**). The spatial proximity between Mfn2 and Plin5 proteins was further validated by immunofluorescence staining and PLA, and Mfn2-Plin5 tethering in CIH+MPP^+^ treated neurons was significantly reduced (**Figure 6E-F**). Collectively, these findings indicate that Mfn2-Plin5 tethering in DA neurons were impaired under CIH+MPP^+^ conditions.

**Figure 7:**
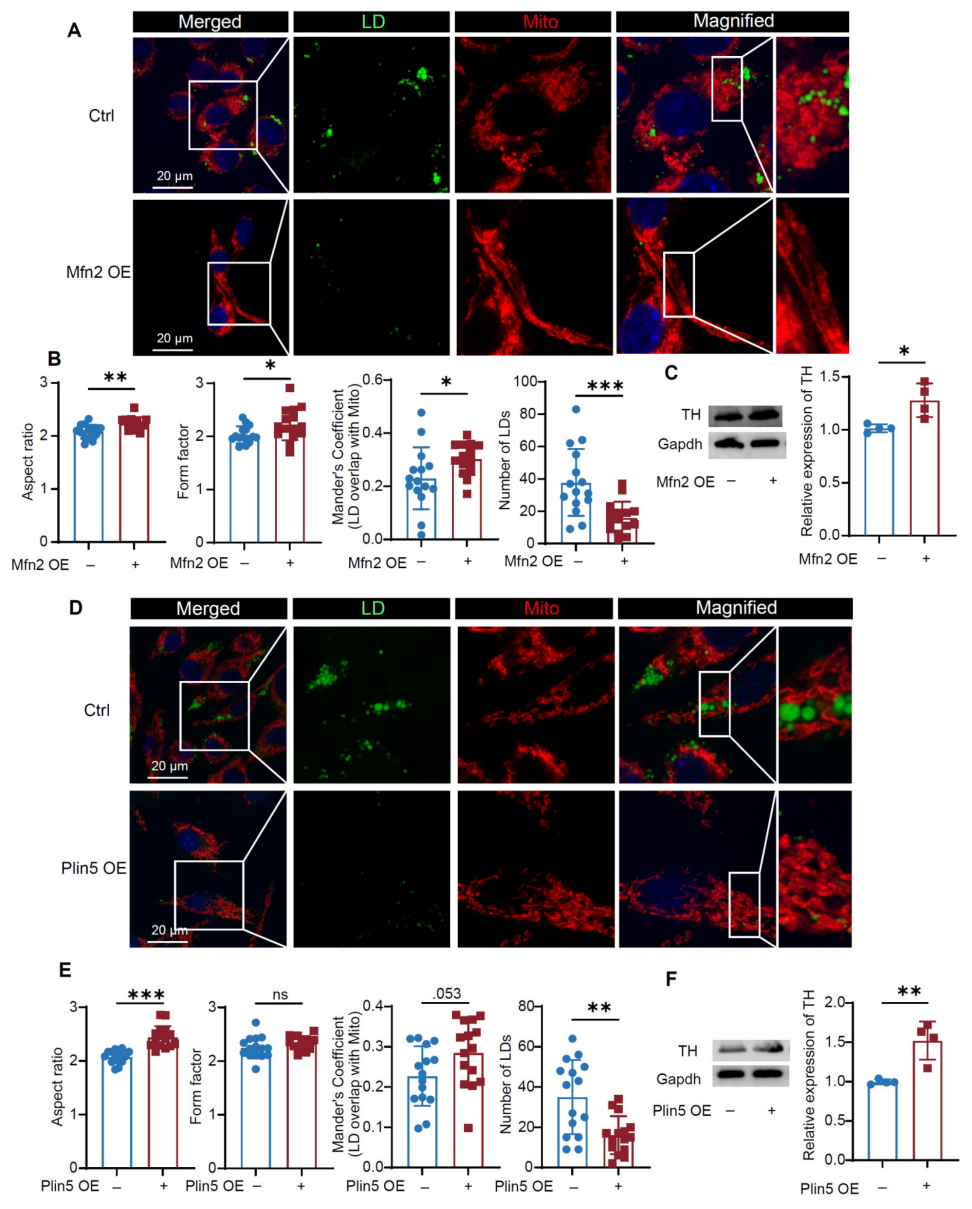
Mfn2/Plin5 overexpression rescues the LD-mitochondrial contacts in DA neurons. **(A)** Representative immunofluorescence images of LD and mitochondria staining in MN9D. (**B**) Statistical analysis of the AR, FF of mitochondria, Mander's coefficient between LDs and mitochondria and relative number of LDs. n = 15 cells from 3 independent experiments. (**C**) Expression of TH in MN9D cells in different conditions. n = 4 independent experiments. **(D)** Representative immunofluorescence images of LD and TOMM20 staining in MN9D cells. (**E**) Statistical analysis of the AR, FF of mitochondria, Mander's coefficient between LDs and mitochondria and relative number of LDs. n = 15 cells from 3 independent experiments. (**F**) Expression of TH in MN9D cells in different conditions. n = 4 independent experiments. Two-tailed Student's t-test was applied for all experiments. ns = no significant, * *p*<0.05, ** *p*<0.01, *** *p*<0.001.

To further evaluate the role of Mfn2-Plin5 in mediating LD-mitochondrial interactions in DA neurons, we overexpressed Mfn2 or Plin5 in MN9D cells (**Figure S9F**). Mfn2 overexpression markedly attenuated mitochondrial fragmentation, significantly increased LD-mitochondrial colocalization, and decreased LD content in DA neurons (**Figure 7A, B**). Similarly, Plin5 overexpression robustly decreased LD content and promoted LD-mitochondrial coupling (**Figure 7D, E**). Importantly, both Mfn2 and Plin5 overexpression upregulated TH expression (**Figure 7C, F**), providing direct evidence that overexpressing Mfn2/Plin5 alleviated impaired LD-mitochondrial interactions and protected DA neurons against CIH+MPP^+^-induced injury. These data identify the Mfn2-Plin5 complex as a core molecular tether mediating LD-mitochondrial interactions, whose disruption under CIH+MPP^+^ conditions contribute to the observed impairment of LD-mitochondrial coupling in DA neurons.

### PPARα regulates the Mfn2/Plin5 tethering and LD metabolism in DA neurons

**Figure 8:**
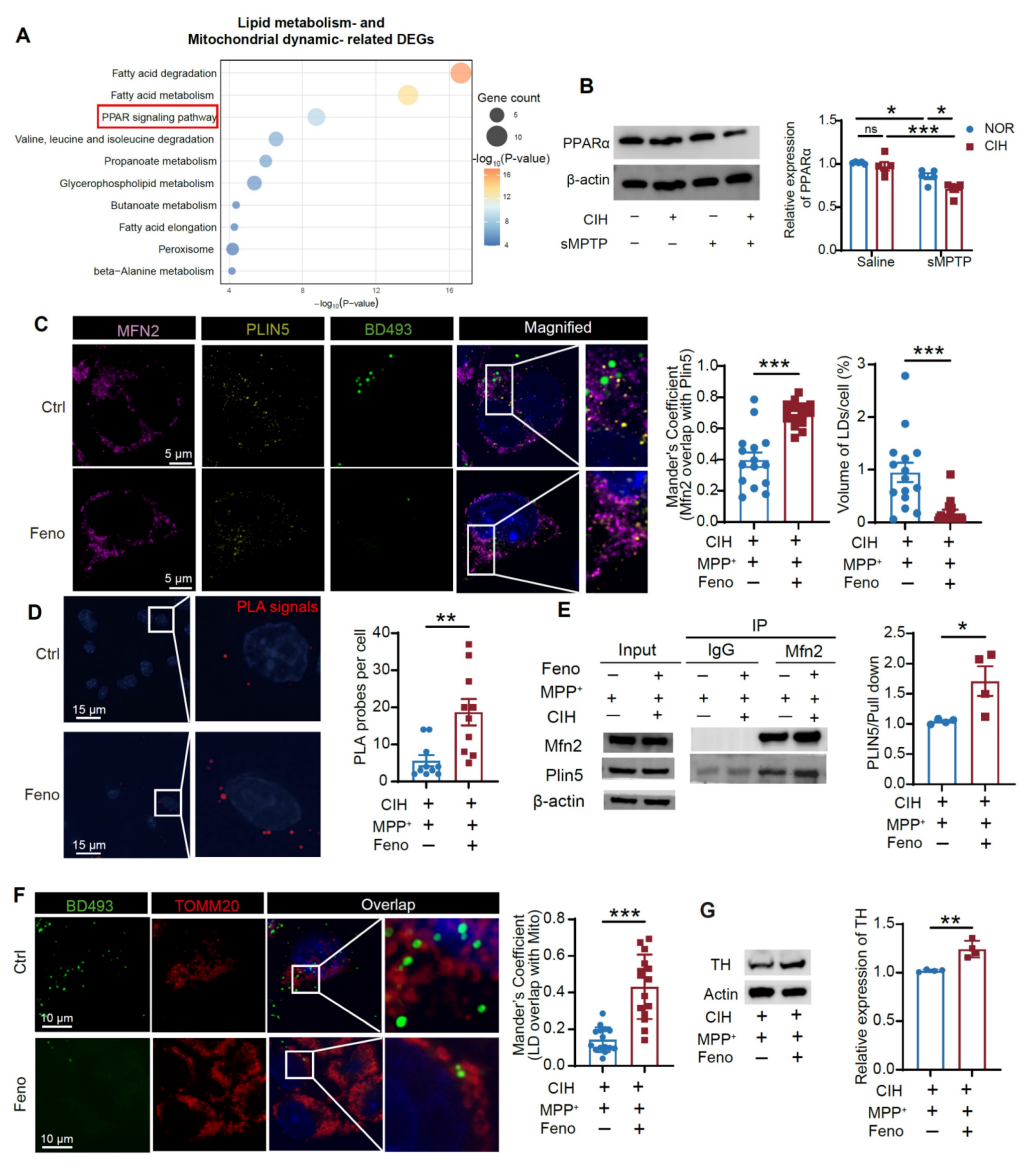
PPARα regulates the Mfn2/Plin5 interaction and the LD-mitochondrial contacts in DA neurons. **(A)** KEGG enrichment analysis of lipid metabolism and mitochondrial dynamics-related DEGs. **(B)** Expression of PPARα in SN in different groups. n = 5 mice for each group. **(C)** Left: Representative immunofluorescence images of Mfn2, Plin5, and LD staining. Right: Statistical analyses of the overlap coefficient between LDs and mitochondria and volume of LDs accumulated in MN9D cells. n = 15 cells randomly selected from 3 independent experiments. **(D)** Representative images and quantitative analysis of PLA signals in MN9D cells. PLA signals are visible as red dots. n = 10 cells randomly selected from 3 independent experiments in each group. **(E)** Co-IP assay using Mfn2 antibody to determine the binding of Mfn2 and Plin5, and statistical analysis of fold change of plin5 and plin2 pulled down. n = 4 independent experiments. **(F)** Representative immunofluorescence images of LD and TOMM20 staining, and statistical analysis of the overlap coefficient between LDs and mitochondria in MN9D cells across different groups, n = 15 cells randomly selected in each group. **(G)** TH expression in different groups. n = 4 independent experiments. Two-way ANOVA with Tukey's post hoc multiple comparisons test for PPARα expression analysis. Mann-Whitney test for overlap coefficient analysis of LD-mitochondrial colocalization. Two-tailed Student's t-test for other tests. ns = no significant, * *p* < 0.05, ** *p* < 0.01, *** *p* < 0.001.

To elucidate mechanisms governing Mfn2 and Plin5 tethering and LD-mitochondrial interaction, we conducted a KEGG pathway analysis on DEGs related to lipid metabolism and mitochondrial dynamics. This revealed significant enrichment in the "PPAR signaling pathway" (KEGG map03320) (**Figure 8A; Figure S10A**). Consistently, PPARα expression was significantly reduced in the CIH+sMPTP treated mice and CIH+MPP^+^ treated MN9D cells (**Figure 8B; Figure S10B**). We next employed fenofibrate, an FDA-approved PPARα agonist with favorable safety, to assess functional consequences 38. Fenofibrate treatment elevated PPARα, Mfn2, and Plin5 protein levels in CIH+MPP^+^ -treated MN9D cells (**Figure S10C**). Using the JASPAR and UCSC databases, we identified putative PPARα-binding sites within the promoter regions of Mfn2 and Plin5 (**Figure S10D-E**), which further supports a direct regulatory role of PPARα and are in line with previous studies demonstrating direct transcriptional regulation of Mfn2 and Plin5 by PPARα 39,40. At the functional level, fenofibrate significantly enhanced Mfn2-Plin5 colocalization by immunofluorescence (**Figure 8C**), increased PLA signals (**Figure 8D**), and elevated Plin5-Mfn2 interaction (**Figure 8E; Figure S10F**). Furthermore, fenofibrate enhanced LD-mitochondrial colocalization in DA neurons (**Figure 8F**), accompanied by increased TH expression in MN9D cells treated with CIH+MPP^+^, indicating improved neuronal function (**Figure 8G**).

**Figure 9:**
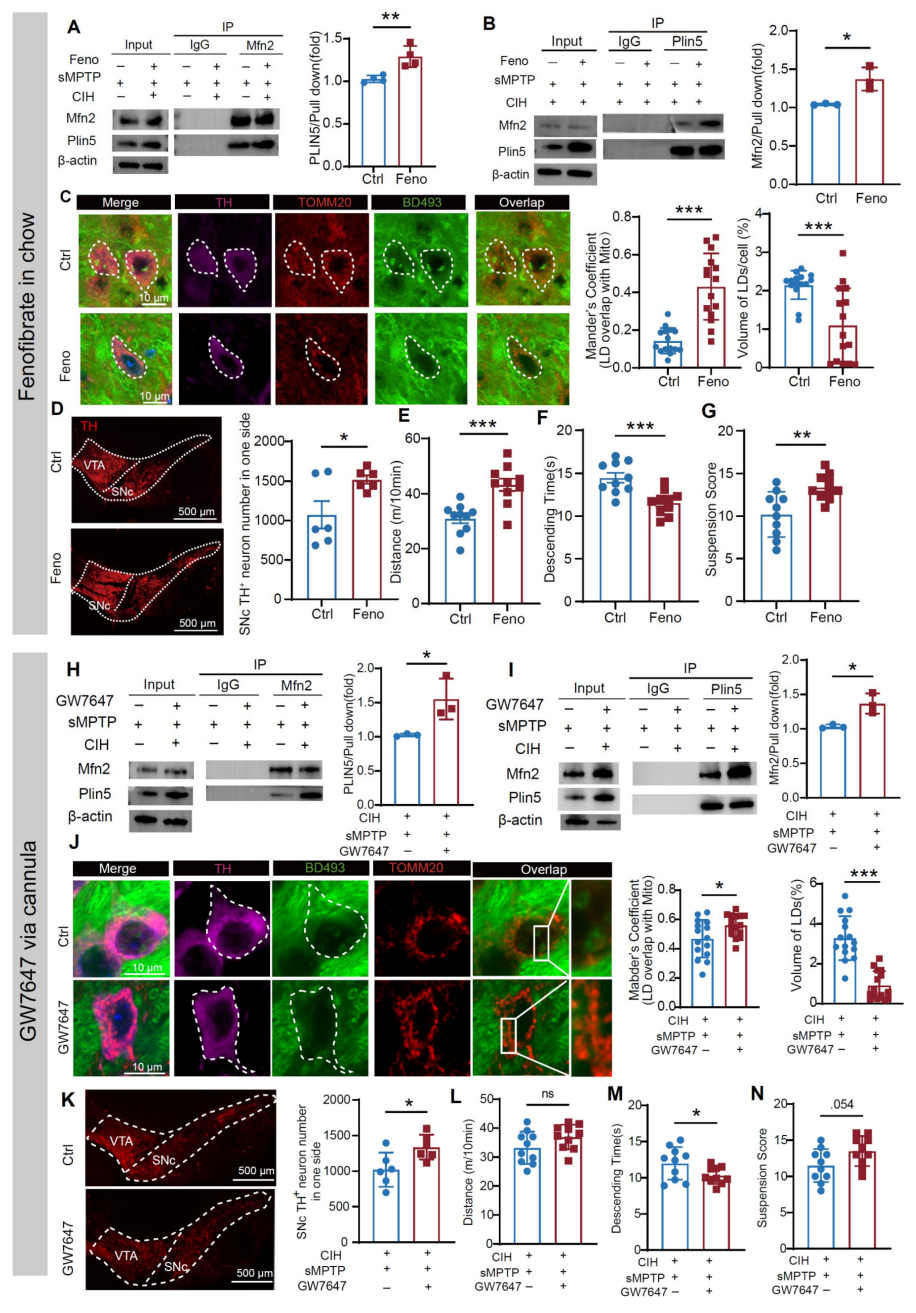
PPARα agonists elevate Mfn2/Plin5 interaction and LD-mitochondrial contacts to alleviate CIH-induced PD susceptibility. **(A)** Co-IP assay using Mfn2 antibody to determine the change of Mfn2 and Plin5 binding in SN of various groups. n = 4 independent experiments. **(B)** Co-IP assay using Plin5 antibody to determine the change of Mfn2 and Plin5 binding in SN of different groups. n = 3 independent experiments. **(C)** Left: Representative images of LD and TOMM20 immunofluorescence staining. Right: Statistical analyses of the overlap coefficient between LDs and mitochondria and the relative volume of LDs in SNc DA neurons in different groups. n = 15 cells from 4 mice. **(D)** Representative images and statistical analysis of the number of SNc TH^+^ cells in different groups, n = 6 mice. **(E-G)** Statistical analyses of total distance traveled in OFT (**E**), descending time (**F**), and suspension score (**G**) in different groups. n = 10 mice. **(H)** Co-IP assay using Mfn2 antibody to determine the change of Mfn2 and Plin5 binding in SN of different groups. n = 3 independent experiments. **(I)** Co-IP assay using Plin5 antibody to determine the change of Mfn2 and Plin5 binding in SN of different groups. n = 3 independent experiments. **(J)** Left: Representative images of LD and TOMM20 immunofluorescence staining. Right: Statistical analyses of the overlap coefficient between LDs and mitochondria and the relative volume of LDs in SNc DA neurons in different groups. n = 15 cells from 4 mice. **(K)** Representative images and statistical analysis of the number of SNc TH^+^ cells in one side, n = 6 mice for each group. **(L-N)** Statistical analyses of total distance traveled in OFT (**L**), descending time (**M**), and suspension score (**N**). n = 10 mice. Mann-Whitney test for Mander's coefficient analysis and LD volume analysis in (**C**) and LD volume analysis in (**J**). Two-tailed Student's t-test for other comparisons. ns = no significant, * *p*<0.05, ** *p*<0.01, *** *p*<0.001.

We further administered fenofibrate to CIH+sMPTP -treated mice to evaluate the alleviation potential in PD susceptibility exacerbated by CIH (**Figure S11A**). Fenofibrate significantly upregulated PPARα, Mfn2, and Plin5 protein levels compared to the CIH+sMPTP controls (**Figure S11B**). Co-immunoprecipitation confirmed enhanced Mfn2-Plin5 interaction following fenofibrate administration (**Figure 9A-B**). Conversely, fenofibrate attenuated the Mfn2-Plin2 binding while reducing Plin2 protein levels (**Figure S11C**). These results further established impaired Mfn2/Plin5, but not Mfn2/Plin2, tethering as the primary mediator of disrupted LD-mitochondrial interactions in DA neurons. Consistent with the *in vitro* findings, fenofibrate enhanced LD-mitochondrial contacts and reduced LD accumulation in SNc DA neurons (**Figure 9C**). The SNc TH^+^ neurons and striatal TH expression were protected by fenofibrate in the CIH-exposed PD-susceptible mice (**Figure 9D; Figure S11D**). Behavioral assessments demonstrated that fenofibrate ameliorated motor deficits, including increased travel distance in the OFT, reduced descending time in the pole test, and higher scores in the hang test (**Figure 9E-G**).

To specifically assess PPARα's role within the SN, we administered another PPARα agonist GW7647 unilaterally into the SN via an implanted cannula (**Figure S11E-F).** Similar to systematically administration of fenofibrate, SN-specific PPARα activation enhanced LD-mitochondrial colocalization and reduced LD accumulation in SNc DA neurons (**Figure 9J**), ameliorated the loss of TH^+^ neurons in SNc (**Figure 9K**), and upregulated TH expression levels in the striatum (**Figure S11G**). Although performance in OFT remained unchanged, GW7647 improved motor function in pole and hanging tests (**Figure 9L-N**). Collectively, these findings suggest that PPARα is a key regulator of LD-mitochondrial crosstalk and lipid metabolism, demonstrating that both systemic (fenofibrate) and nigral-targeted (GW7647) PPARα activation restore lipid homeostasis and mitigate nigrostriatal degeneration in CIH-aggravated PD models.

## Discussion

The association between OSA and PD was reported in clinical studies 13, yet how CIH, the dominant pathological feature of OSA, impacts the PD-related pathology remains poorly understood. In this study, we demonstrate that CIH treatment significantly exacerbates motor deficits and neuropathology across three independent subtoxic PD models. Mechanistically, CIH treatment disrupts PPARα-mediated LD-mitochondrial contacts, thereby impairing LD degradation and promoting pathological LD accumulation in DA neurons. Importantly, activating PPARα not only upregulates the expression of Mfn2 and Plin5 but also promotes Mfn2-Plin5 tethering, re-establishing LD-mitochondrial coupling and facilitating LD catabolism in DA neurons, which ultimately rescues nigrostriatal DA neurodegeneration and alleviates motor deficits in CIH-exposed PD models. Collectively, these findings unveil a previously unrecognized mechanistic link between CIH and PD progression, positioning PPARα-mediated LD-mitochondrial interaction as a central node in the intersection of OSA and PD. This study not only advances our understanding of how CIH contributes to PD pathogenesis but also identifies PPARα as a promising therapeutic target for OSA-associated PD.

OSA, the most prevalent sleep and breathing disorder in older people 41, has been predominantly associated with cognitive deficits and mechanisms of CIH-induced hippocampal neurodegeneration 42. Critically, recent epidemiological and clinical studies establish OSA as a significant risk factor that increases PD incidence and exacerbates pathology in patients. However, the mechanisms underlying CIH-associated PD susceptibility remain poorly defined. Our multi-model study, conducted across three distinct PD mouse models and *in vitro* cellular model, demonstrates that CIH alone does not induce motor dysfunction or nigrostriatal DA degeneration. However, it synergistically exacerbates PD-related phenotypes when combined with subtoxic doses of parkinsonism toxin or pathological protein (Figure 1; Figure S1). These findings align with previous experimental evidence demonstrating that acute hypoxia potentiates motor deficits and dopaminergic neuron loss in PFF-injected models without causing behavioral alterations 43. The unique vulnerability of SNc DA neurons likely stems from their exceptional bioenergetic demands, driven by extensively arborized axons, abundant presynaptic active zones, and pacemaker-like autonomous activity—features rendering them exquisitely sensitive to mitochondrial dysfunction and hypoxic stress 44. These findings indicate that early therapeutic management of sleep-disordered breathing is a clinically actionable strategy for mitigating PD risk.

It is becoming more well acknowledged that lipid dyshomeostasis is a fundamental pathogenic characteristic of neurodegenerative diseases. Our integrated bulk sequencing and lipidomic analyses identified perturbations in lipid metabolism as a critical mechanism underlying CIH-induced PD susceptibility (Figure 2; Figure S2). Across both* in vitro* and* in vivo* PD models, we observed CIH-triggered LD accumulation in DA neurons and microglia, which recapitulates LD pathology in postmortem PD brains 45. Notably, neither CIH alone nor subtoxic PD-related insults independently induced LD pathological accumulation, whereas their combination exerted a synergistic effect that amplifies lipid metabolic dyshomeostasis. Furthermore, lipid-overloaded DA neurons promoted microglial LD accumulation via intercellular lipid transfer (Figure S5). While CIH may directly modulate microglial lipid-handling pathways, our co-culture experiments indicate that DA neuron-derived lipids induce microglial LD accumulation through an APOE-dependent mechanism. Meanwhile, microglia with accumulated LDs adopted a proinflammatory phenotype that exacerbated DA neuronal injury (Figure 3). This observation aligns with neuron-to-microglia lipid trafficking in hippocampal contexts 29. Critically, enhancing lipid catabolism to reduce LD accumulation significantly improved DA neuronal survival and ameliorated motor deficits in CIH-exposed PD-susceptible mice (Figure 5). This protection likely involves ATGL recruitment to mitochondrial interfaces during LD mobilization, enabling direct channeling of liberated FAs into mitochondria for β-oxidation, thereby boosting cellular energetics while avoiding lipotoxicity 46. These findings highlight LD deposition and metabolism as promising therapeutic targets for mitigating CIH-associated PD pathology.

Membrane contact sites between LDs and mitochondria critically regulate LD metabolism and cellular functions 47. Our study extends this regulatory paradigm to PD pathology, revealing that CIH impairs LD-mitochondrial interactions in DA neurons (Figure 4). Pharmacological promotion of lipolysis or suppression of mitochondrial fragmentation effectively restored LD-mitochondrial communication, reduced LD deposition, and ameliorated motor deficits and neurodegeneration in PD-susceptible mice (Figure 5). Rambold et al. also demonstrated that inhibiting mitochondrial fusion dissociates LDs from mitochondria during nutrient deprivation, impairing fatty acid oxidation and promoting LD accumulation 35. Conversely, studies in brown adipocytes show that LD-mitochondrial tethering promotes FA esterification and LD expansion 48. This functional dichotomy reflects tissue-specific metabolic demands: energy-storing tissues (e.g., adipose) utilize contacts for lipid sequestration, while energy-demanding tissues (e.g., brain and heart) require tethering for efficient LD mobilization to sustain ATP production.

Mechanistically, LD-mitochondrial tethering is mediated by protein interactions between organelle-anchored proteins, with distinct complexes operating in a tissue-, condition-, and function-dependent manner 49. Our results specifically identify disruption of Mfn2-Plin5 tethering as the basis underlying reduced LD-mitochondrial contacts in DA neurons (Figure 6). Overexpression of Mfn2 or Plin5 effectively restored LD-mitochondrial interactions (Figure 7). Although we observed increased Mfn2-Plin2 binding following both CIH treatment and fenofibrate exposure reversed this increase (Figure S8; Figure S10), it has been documented that overexpression of full-length Plin2 alone will not influence LD-mitochondrial contacts, but fusion of the C-terminal domain of Plin5 to Plin2 will elevate LD anchoring to mitochondria 50. Thus, while Mfn2-Plin2 binding is elevated in our CIH model, it appears insufficient to support functional LD-mitochondrial coupling, highlighting the specific role of Mfn2-Plin5 tethering in maintaining LD-mitochondrial dynamics in DA neurons.

The PPAR family orchestrates transcriptional regulation of critical cellular processes such as inflammation, energy homeostasis, and lipid/glucose metabolism 51. PPARα agonists have been demonstrated to exert neuroprotective effects in PD by encompassing the maintenance of glutamate homeostasis, suppression of inflammation, and attenuation of gliosis 52. Consistently, our study reveals that DA neurodegeneration and motor deficits in CIH-induced PD-susceptible mice were reversed by PPARα agonists fenofibrate and GW7647 (Figure 8, 9). Beyond its established role as a master transcriptional factor for lipid metabolic enzymes like CPT1A, PPARα can modulate *MFN2* and *PLIN5* transcription (Figure S9) 39,40. Importantly, our findings extend the role of PPARα in regulating lipid metabolism, as our results demonstrated that PPARα activation not only upregulated Mfn2 and Plin5 protein levels but also enhanced their physical interaction, thereby promoting LD-mitochondrial tethering and reducing pathological LD accumulation in DA neurons. Collectively, these findings highlight PPARα-mediated LD-mitochondrial dynamics as a pivotal link between CIH and PD pathogenesis and underscore its potential as a clinically actionable target for alleviating PD vulnerability in patients with OSA.

Our study demonstrates that CIH disrupts PPARα-mediated LD-mitochondrial dynamics, leading to LD metabolic abnormalities and exacerbation of PD-related pathology. Pharmacological activation of PPARα, inhibition of excessive mitochondrial fission, or alleviation of LD degradation defects effectively blocked this pathological cascade and mitigated CIH-induced PD susceptibility, highlighting potential therapeutic targets for reducing PD risk in patients with OSA. However, it is important to recognize a number of this study's limitations. First, we found that lipid transfer between DA neurons and microglia was primarily mediated by APOE, as competitive inhibition of LDLR reduced lipid transfer by more than 50% (Figure 3). However, intercellular lipid trafficking is not restricted to the APOE pathway. Previous studies have reported that tunneling nanotubes (TNTs) and secretory vesicles, including exosomes and microvesicles, also participate in lipid transfer between cells 53,54. In this study, we did not evaluate the contribution of these additional pathways and future investigations should systematically assess their roles by employing microtubue inhibitors (e.g., vincristine) to disrupt TNTs formation or exosome secretion inhibitors (e.g., GW4869) in combination with lipid tracing assays. Secondly, although PPARα is known to regulate transcription by directly binding to the promoters of target genes, it can also act through non-geomic mechanisms, such as suppressing the activity of other transcription factors (e.g., NF-κB) 55. In the current study, we did not determine whether the regulation of Mfn2 and Plin5 by PPARα is mediated through direct promoter binding or indirect signaling pathways. Future studies should address this by performing ChIP-qPCR or ChIP-seq to directly validate PPARα binding to the Mfn2 and Plin5 promoters, and by incorporating luciferase reporter assays to further clarify the transcriptional regulatory mechanisms.

## Conclusion

We have demonstrated that CIH increases the susceptibility to PD by exacerbating the motor dysfunction and neuropathology in various subtoxic PD models. We have shown that the interaction between LDs and mitochondria plays a pivotal role in protecting against PD by promoting LD degradation and reducing LD accumulation in DA neurons. Furthermore, we have identified two key proteins, Mfn2 and Plin5, as the molecular mediators for LD-mitochondrial interaction. PPARα was identified as a target for enhancing the tethering of Mfn2-Plin5, thus modulating LD-mitochondrial interaction and reducing LD levels. Activation of PPARα, whether systematically or locally, has shown significant neuroprotective effects, suggesting its promising potential for PD intervention. Our findings not only underscore the importance of LD-mitochondrial dynamics as a critical vulnerability node in the comorbidity of OSA and PD, but also highlight lipid metabolic modulation as a promising therapeutic strategy for neurodegenerative disorders.

## Supplementary Material

Supplementary figures.

## Abbreviations

ACC1: acetyl-CoA carboxylase 1
APOE: apolipoprotein E
ATGL: adipose triglyceride lipase
α-syn: α-synuclein
CE: cholesteryl ester
CIH: chronic intermittent hypoxia
CM: conditioned medium
CNS: central nervous system
Co-IP: co-immunoprecipitation
DA: dopaminergic
DEG: differentially expressed genes
FA: fatty acid
GO: gene ontology
LD: lipid droplet
LDL: low-density lipoprotein
Mfn2: mitofusin-2
OFT: open field test
OSA: obstructive sleep apnea
PD: Parkinson's disease
PFA: paraformaldehyde
PLA: proximity ligation assay
Plin5: perilipin-5
PPARα: peroxisome proliferator-activated receptor α
RT: room temperature
SNc: substantia nigra pars compacta
TG: triglycride
TH: tyrosine hydroxylase
TNTs: tunneling nanotubes
