# Supplementary material for: Chronic intermittent hypoxia increases Parkinson's disease susceptibility via PPARα-mediated lipid droplet-mitochondrial dysfunction
Source: Theranostics. 2026 Jan 1;16(5):2466–87. doi: 10.7150/thno.122944 (PMC12712921; doi:10.7150/thno.122944)
Supplement: Supplementary file 1 — Supplementary figures. [file thnov16p2466s1.pdf]

Chronic intermittent hypoxia increases Parkinson's disease susceptibility via PPARα-mediated lipid droplet-mitochondrial dysfunction

Ming-Rui Zhai[1, 2], Jie Pan[1, 2], Zhen-Huan Wu[1], Yuying He[1], Kai-Run Zhang[1], Long Ren[1], Yu-Rong Wang[1], Yi-Bing Li[1], Jun Gao[1]***, Lei Xiao[1] **, Yue-Hua Liu[1, 3]*

Table of contents

Fig.S1 2

Fig.S2 4

Fig.S3 5

Fig.S4 6

Fig.S5 8

Fig.S6 10

Fig.S7 11

Fig.S8 12

Fig.S9 14

Fig.S10 16

Fig.S11 18

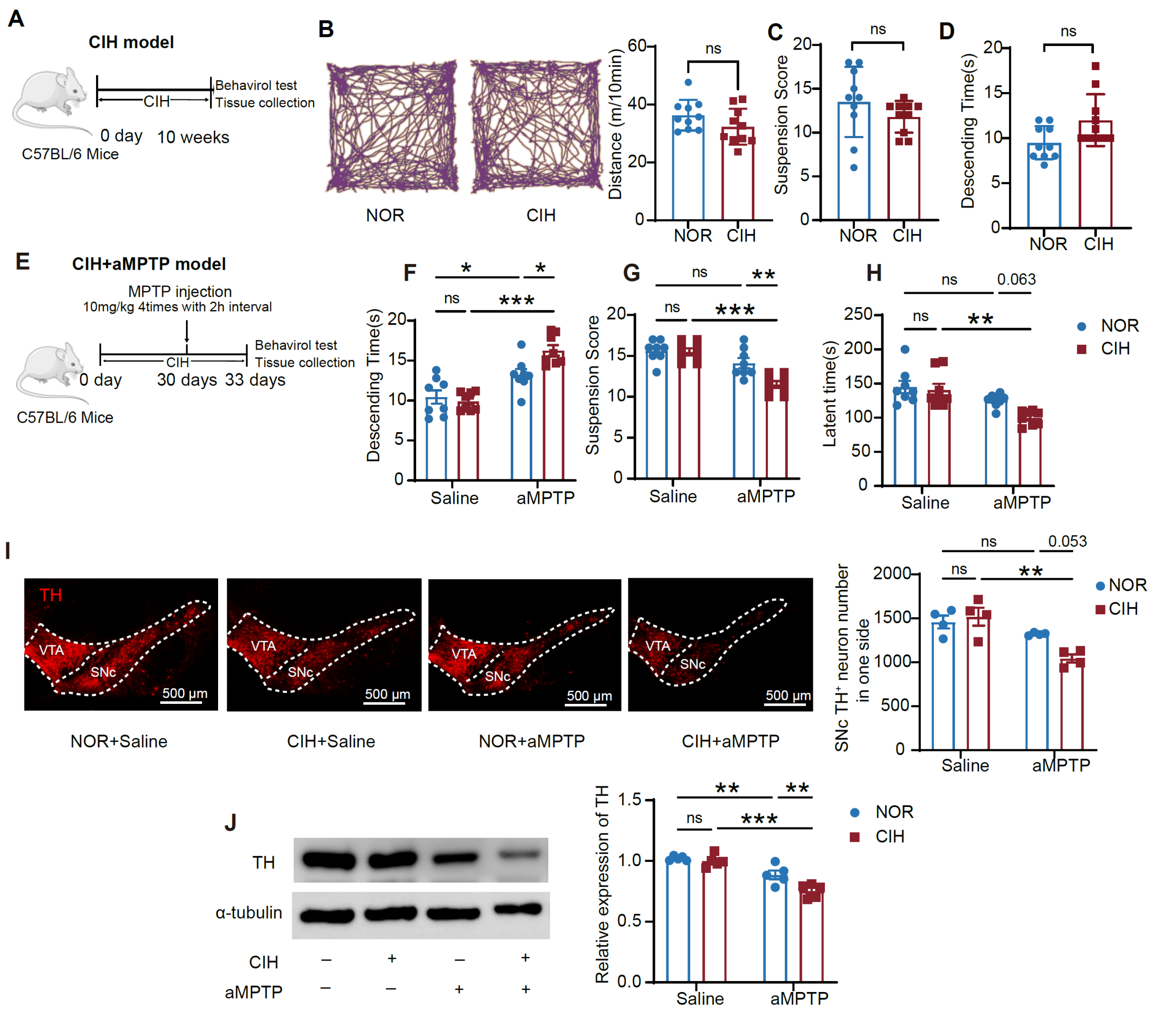

Figure S1 The impacts of CIH and CIH+acute subtoxic MPTP on mouse motor deficits and nigrostriatal DA degeneration. **(A)** Schematic diagram of CIH exposure protocol. **(B)** Example trajectories and statistical analysis of total distance traveled in OFT, n = 10 mice in different groups. **(C-D)** Statistical data of suspension score in the hang test (**C**) and descending time in the pole test (**D**), n = 10 mice in different groups. (**E**) Schematic diagram of CIH+aMPTP exposure protocol. **(F-H)** Descending time spent on the pole (**F**), suspension score (**G**) in hang test, and time spent on the rotarod (**H**). n = 8 mice in each group. **(I)** Representative images and statistical analysis of TH^{+} cells in one SNc side, n = 4 mice in different groups.

(J) Expression levels of TH in the striatum. n = 5 mice in different groups. Two-tailed Student's t-test for OFT between NOR and CIH groups (**B**). Mann-Whitney test for the hang test and pole test between NOR and CIH groups (**C-D**). Two-way ANOVA with Tukey's post hoc multiple comparisons test for other comparisons. ns = no significant, * $p < 0.05$, ** $p < 0.01$, *** $p < 0.001$.

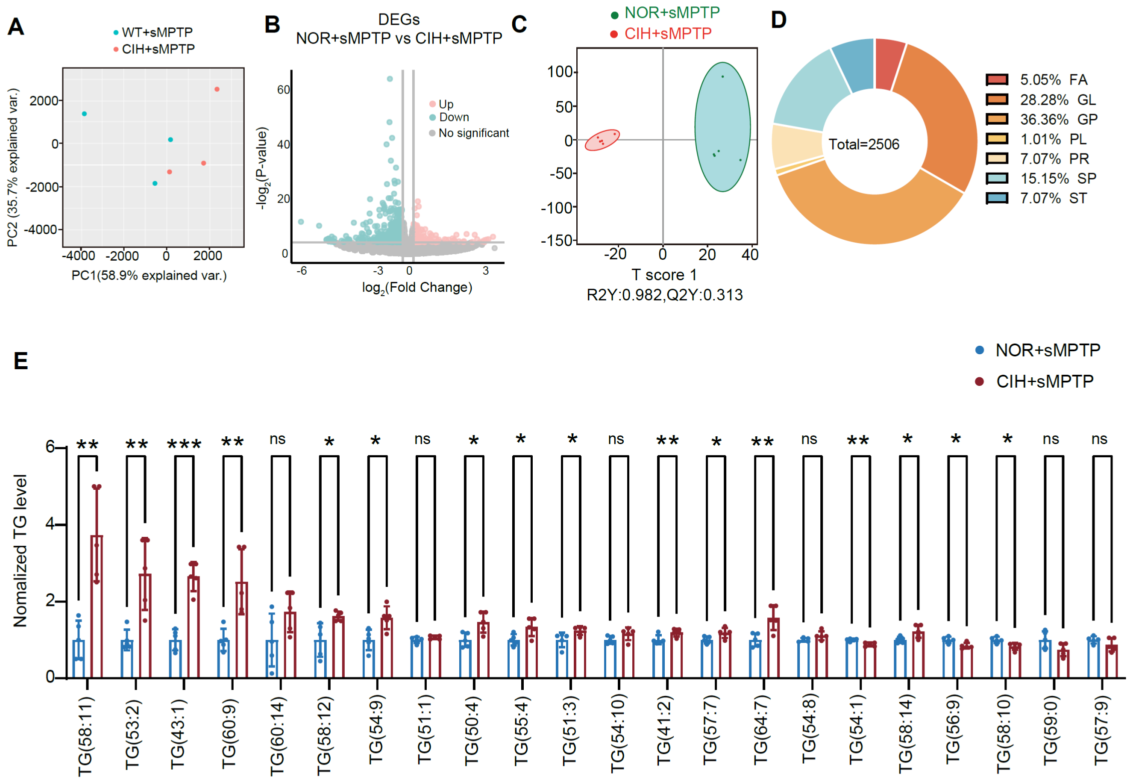

Figure S2 CIH elevates lipid levels in subtoxic PD mouse models. **(A)** PCA clustering analysis between NOR+sMPTP and CIH+sMPTP treated group. n = 3 mice in different groups. **(B)** Volcano plot of DEGs between NOR+sMPTP and CIH+sMPTP treated groups. n = 3 mice in different groups. **(C)** The orthogonal squares discrimination analysis score map based on untargeted high-throughput analysis of lipid profile in the midbrain of NOR+sMPTP and CIH+sMPTP treated mice. n = 5 mice in different groups. **(D)** Number of lipid types identified in untargeted high-throughput analysis. **(E)** Statistical analysis of the triacylglycerol level according to untargeted high-throughput analysis. n = 5 mice in different groups. Two-tailed Student's t-test for all comparisons. ns = no significant, * $p < 0.05$, ** $p < 0.01$, *** $p < 0.001$.

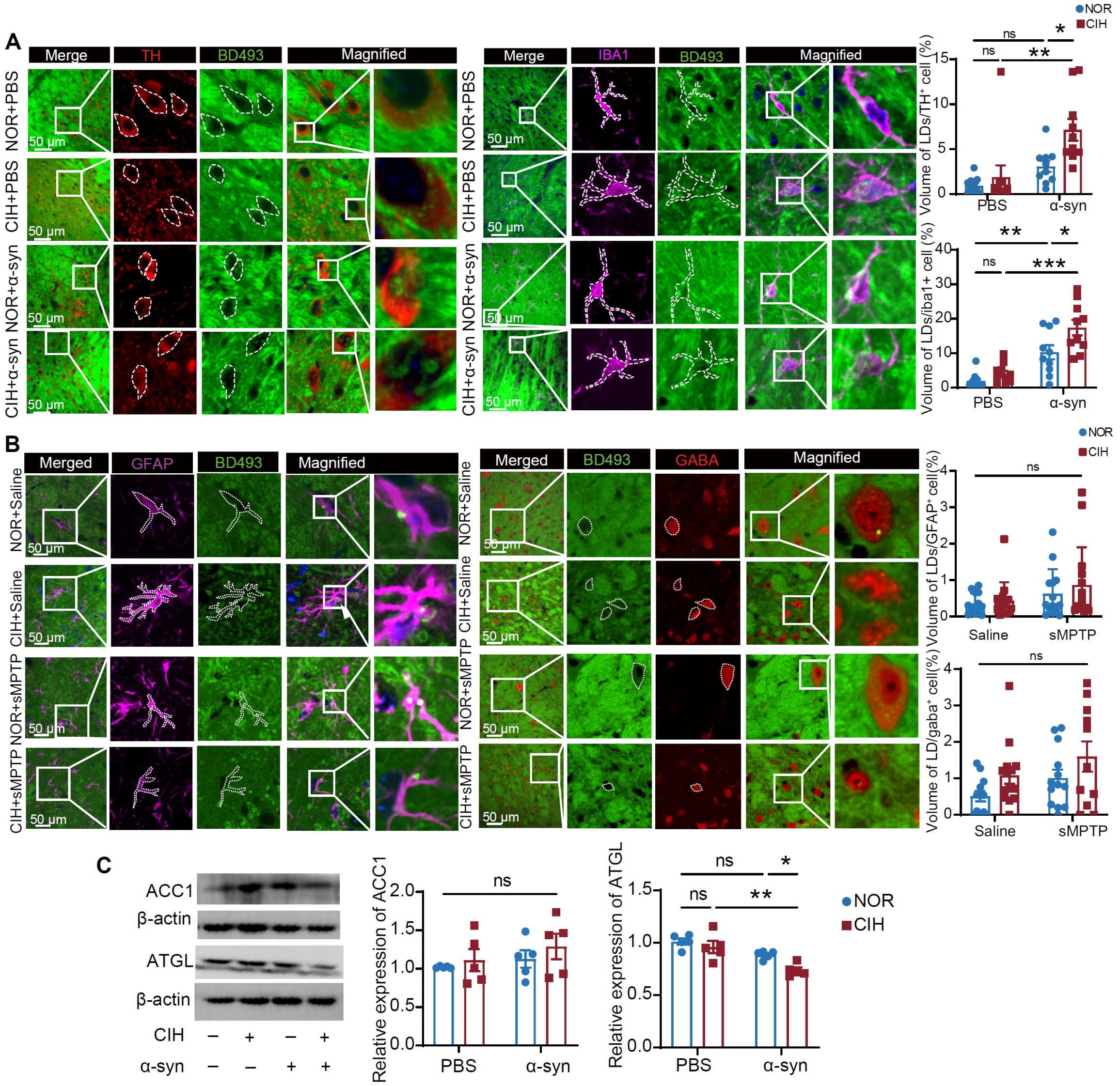

Figure S3 CIH induces cell-specific LD accumulation in subtoxic PD models. **(A)** Representative immunofluorescence images of TH, Iba1, and LD staining in SNc and statistical analyses of LD volume in subtoxic α-syn mouse model. n = 10 cells from 3 mice. **(B)** Representative immunofluorescence images of GFAP, GABA, and LD staining in SNc and statistical analyses of LD volumes in subtoxic MPTP model. n = 15 cells from 3 mice. **(C)** Expression levels of ACC1 and ATGL in SN in subtoxic α-syn model. n = 5 mice in each group. Two-way ANOVA with Tukey's post hoc multiple comparisons test for all comparisons. ns = no significant, * $p < 0.05$, ** $p < 0.01$, *** $p < 0.001$.

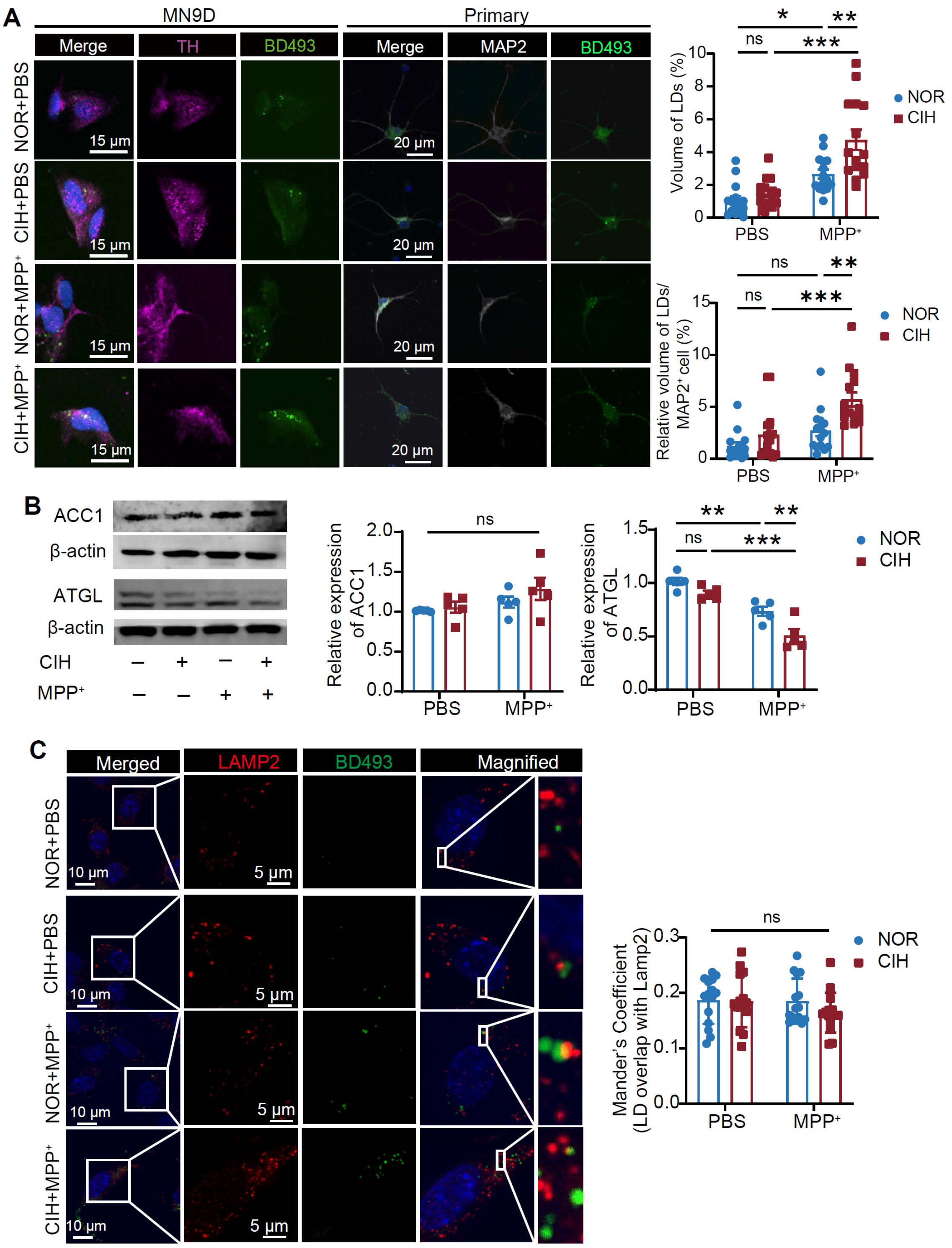

Figure S4 CIH disrupts LD metabolism in cultured MN9D cells. **(A)** Left: Representative images of TH and LD immunofluorescence staining and statistical analysis of LD volume in MN9D cells in different conditions. Right: Representative images of MAP2 and LD immunofluorescence staining and statistical

analysis of LD volume in primary midbrain neurons in different conditions. n = 15 cells from 3 independent experiments. **(B)** Expression levels of ACC1 and ATGL in MN9D cells. n = 5 independent experiments. **(C)** Representative images of LAMP2 and LD immunofluorescence staining and statistical analysis of Mander's coefficient between LD and LAMP2 in MN9D cells. n = 15 cells from 3 independent experiments randomly selected. Two-way ANOVA with Tukey's post hoc multiple comparisons test for all comparisons. ns = no significant, * $p < 0.05$, ** $p < 0.01$, *** $p < 0.001$.

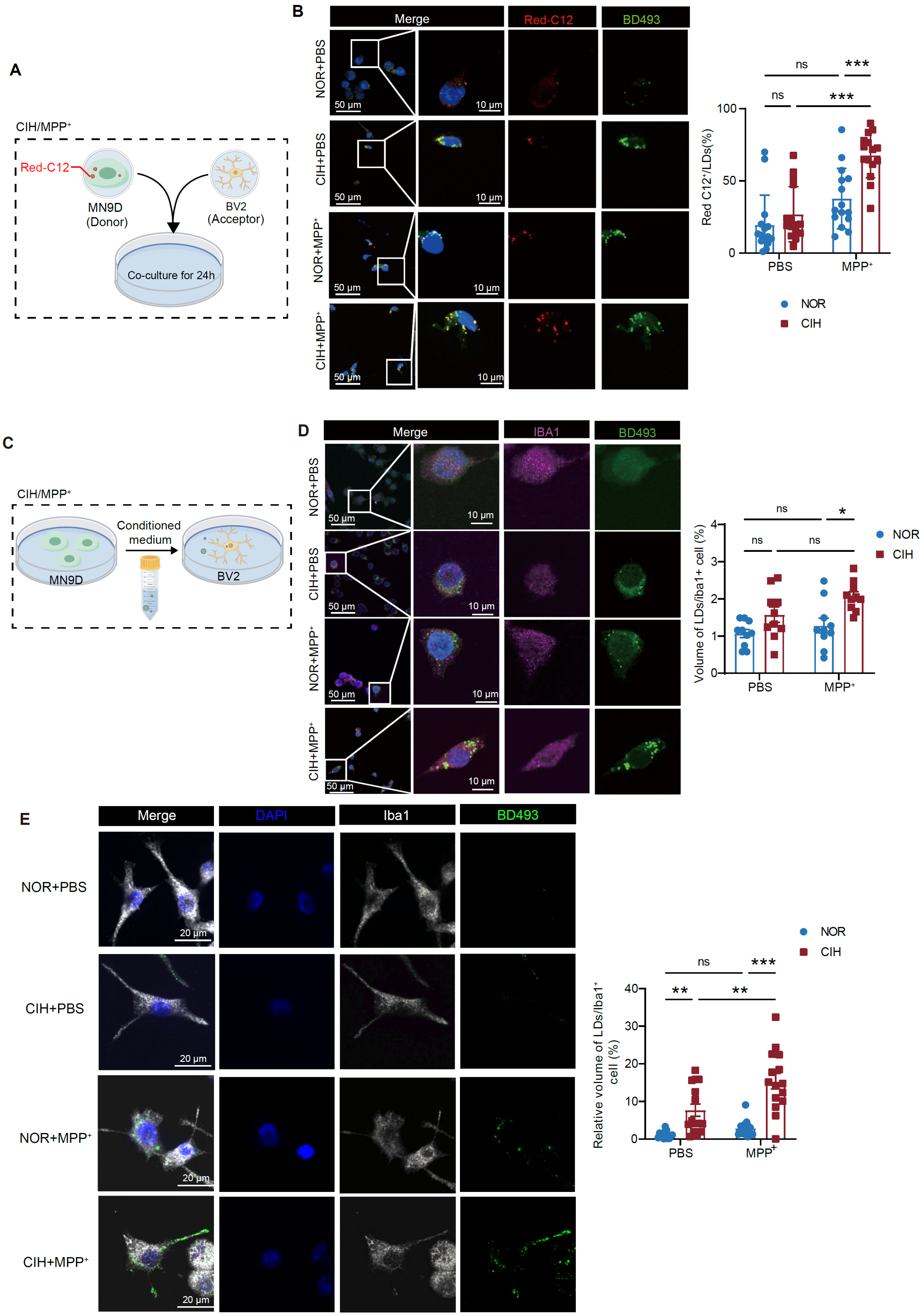

Figure S5 Lipids within DA neurons could be transferred to microglia. **(A)** Scheme representation of

pulse-labeling with Red-C12 in MN9D and BV2 cells. **(B)** Representative images of Red-C12 and LD immunofluorescence staining in BV2 and statistical analysis of the ratio of Red-C12 to LD. n = 15 cells from 3 independent experiments randomly selected. **(C)** Scheme representation of the indirect coculture of MN9D and BV2 cells. **(D)** Representative immunofluorescence images of Iba1 and LD staining in BV2 across different groups and statistical analysis of LD volume in microglia. n = 10 cells randomly selected from 3 independent experiments. **(E)** Representative immunofluorescence images of Iba1 and LD staining in primary microglia and statistical analysis of LD volume in primary microglia. n = 15 cells randomly selected from 3 independent experiments. Two-way ANOVA with Tukey's post hoc multiple comparisons test for all comparisons. ns = no significant, * $p < 0.05$, ** $p < 0.01$, *** $p < 0.001$.

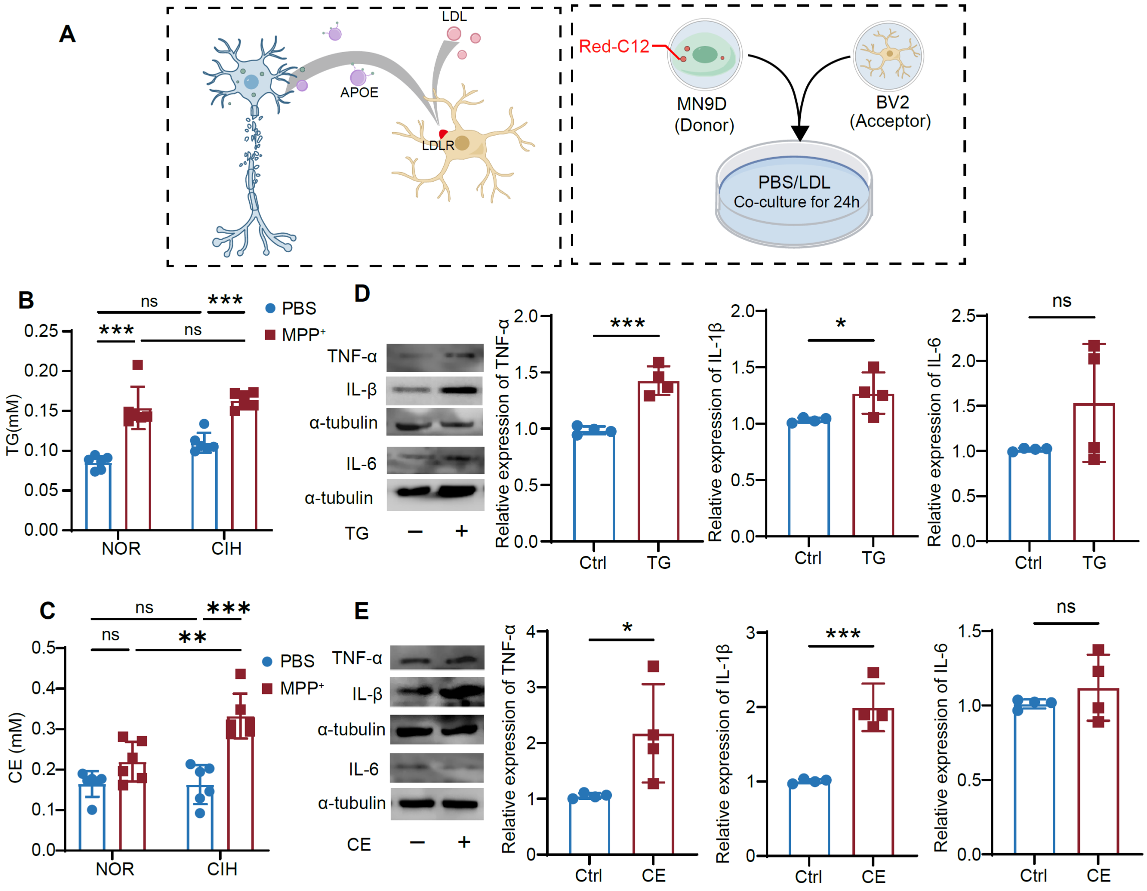

Figure S6 TG and CE induce a pro-inflammatory phenotype of microglia. **(A)** Schematic representation of competitive inhibition of microglial LDLR by LDL and pulse-labeling with Red-C12 in MN9D and BV2 cells. **(B-C)** TG (B) and CE (C) levels in the supernatant of MN9D across different groups. n = 6 independent experiments. **(D-E)** TNF-α, IL-1β, and IL6 expression levels in different conditions. n = 4 independent experiments. Two-tailed Student's t-test for other comparisons. ns = no significant, * $p < 0.05$, ** $p < 0.01$, *** $p < 0.001$.

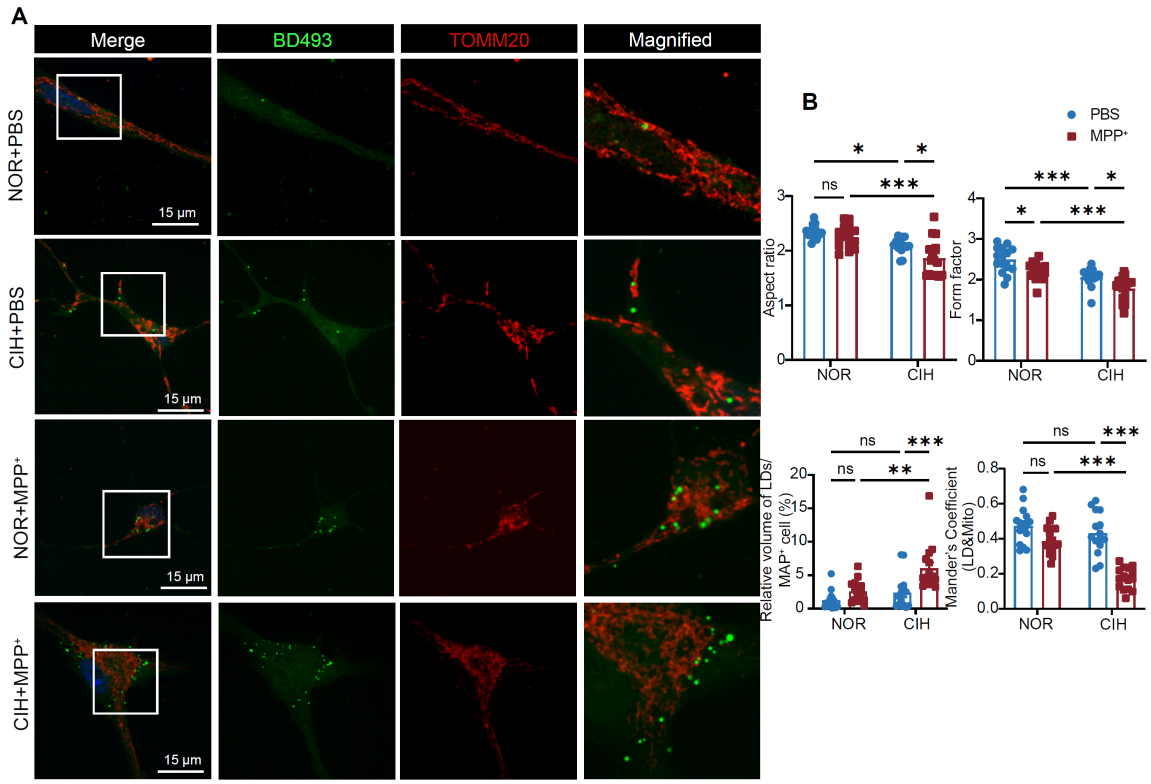

Figure S7 CIH induces mitochondrial fragmentation and impairs LD-mitochondrial interaction in CIH+MPP$^+$ treated primary midbrain neurons. **(A)** Representative immunofluorescence images of MAP2, LD, and TOMM20 staining in primary midbrain neurons. (**B**) Statistical analyses of aspect ratio, form factor, volume of LD in primary midbrain neurons, and overlap coefficient between LD and TOMM20. n = 15 cells from 3 independent experiments. Two-way ANOVA with Tukey's post hoc multiple comparisons test for all comparisons. ns = no significant, * $p < 0.05$, ** $p < 0.01$, *** $p < 0.001$.

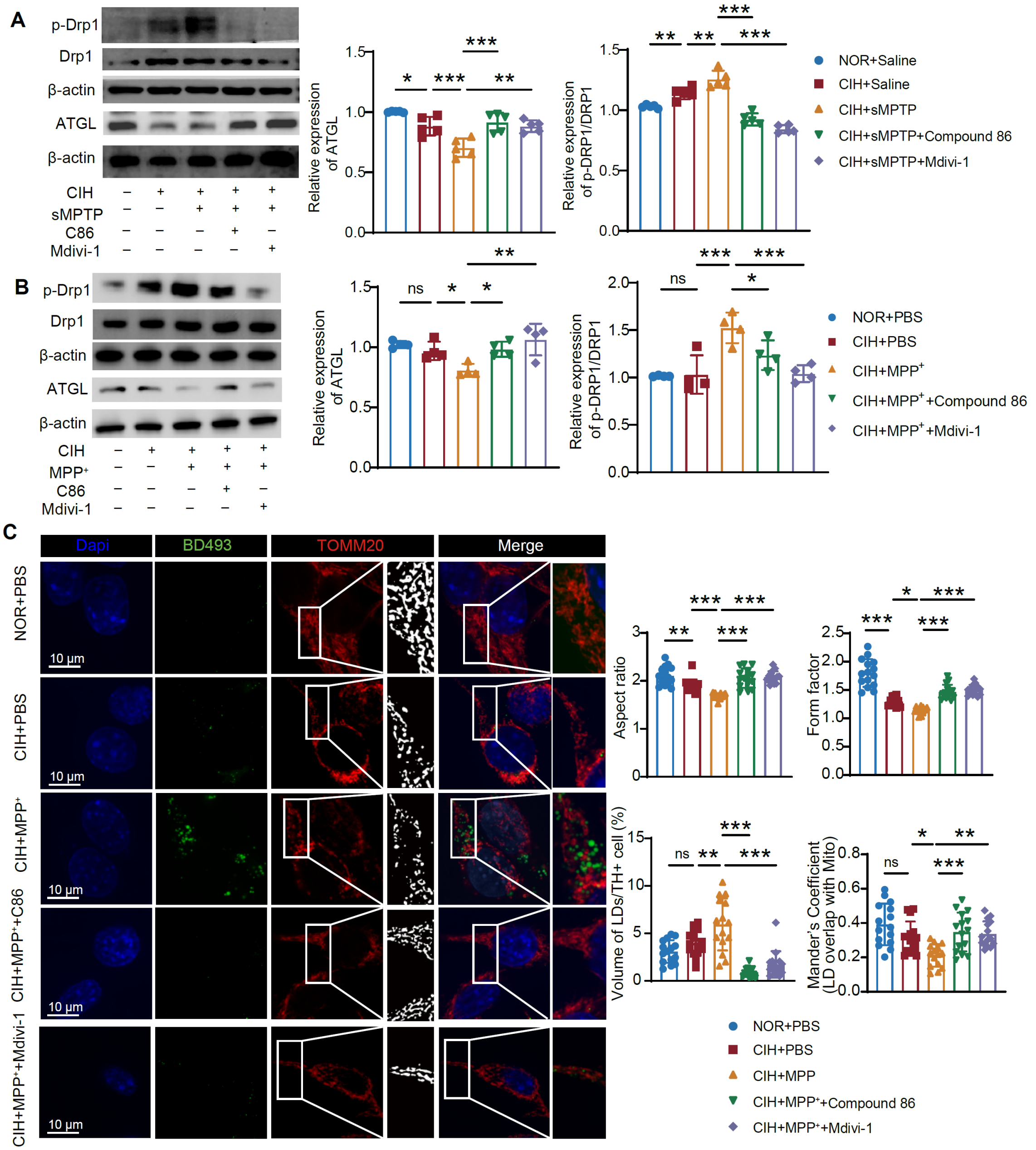

Figure S8 Pharmacological modulation of LD catabolism and mitochondrial fission alleviates LD accumulation and LD-mitochondrial interaction in MN9D cells of the CIH+MPP$^+$ model. **(A)** Expression of ATGL and p-Drp1/Drp1 in SN. n = 5 mice for each group. (**B**)Expression of ATGL and p-Drp1/Drp1 in MN9D cells. n = 4 independent experiments. **(C)** Representative immunofluorescence images of TH, LD, and TOMM20 staining in MN9D cells across different groups. Statistical analysis of aspect ratio, form factor, volume of LD in MN9D, and Mander's coefficient between LD and TOMM20. n = 15 cells from 3

independent experiments. One-way ANOVA with Bonferroni multiple comparisons test for all comparisons.

ns = no significant, * $p < 0.05$, ** $p < 0.01$, *** $p < 0.001$.

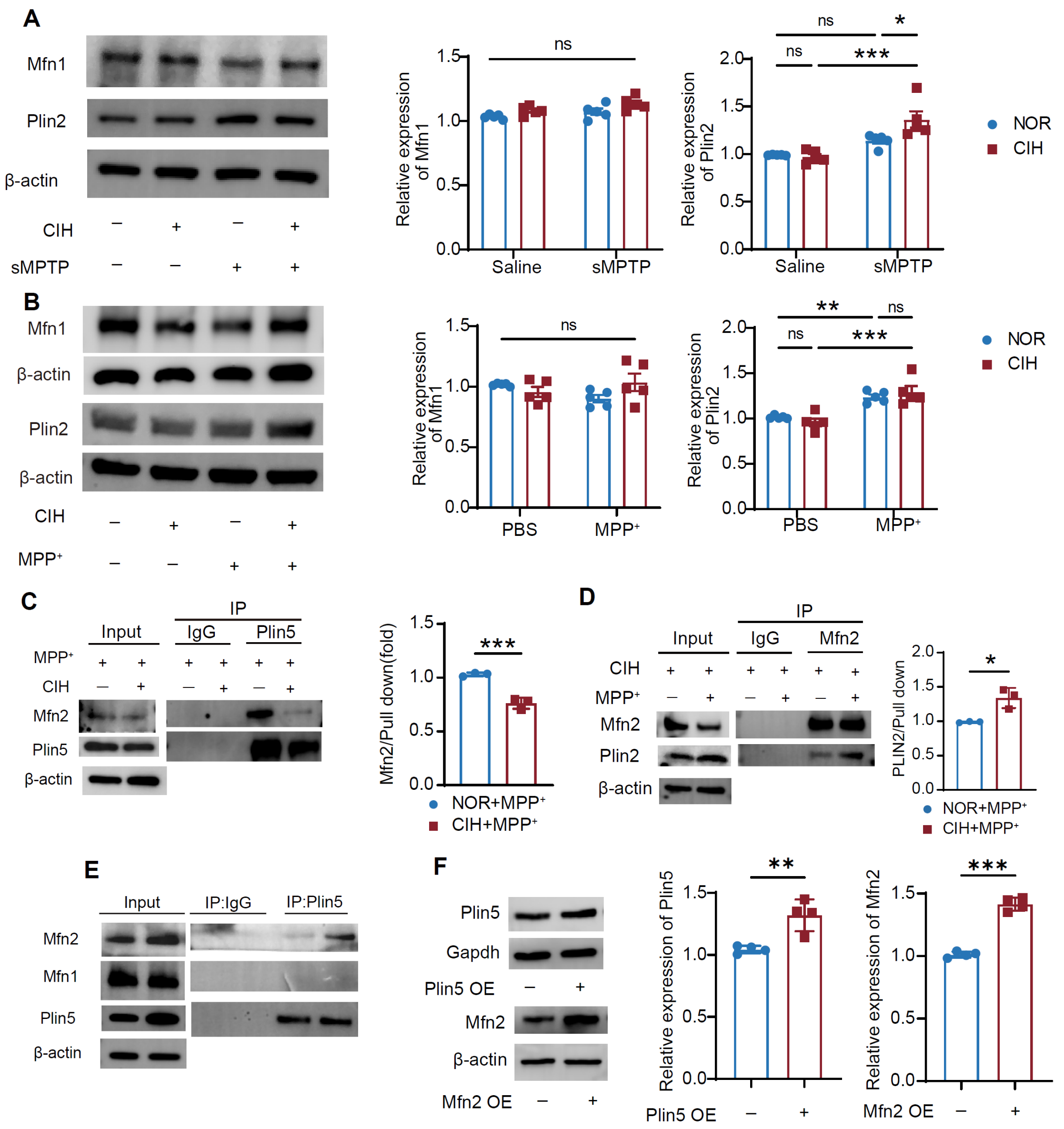

Figure S9 The expression and interaction between Mfn1/2 and Plin2/5 in DA neurons. **(A)** Expression of Mfn1 and Plin2 in SN across different groups. n = 5 mice. **(B)** Expression of Mfn1 and Plin2 in MN9D cells. n = 5 independent experiments. **(C)** Co-IP assay using Plin5 antibody to determine the binding of Mfn2 and Plin5, and statistical analysis of fold change of Mfn2 pulled down. n = 3 independent experiments. **(D)** Co-IP assay using Mfn2 antibody to determine the binding of Mfn2 and Plin2, and statistical analysis of fold change of plin2 pulled down. n = 3 independent experiments. **(E)** Co-IP assay

using Plin5 antibody to determine the binding of Mfn1/2 and Plin5. (**F**) Expression of Mfn2 and Plin5 in MN9D cells. n = 4 independent experiments. Mfn2/Plin2 interaction (**C, D**) and Mfn2/Plin5 expression in (**F**) was analyzed by two-tailed Student's t-test. Two-way ANOVA with Tukey's post hoc multiple comparisons test for other comparisons. ns = no significant, * $p < 0.05$, ** $p < 0.01$, *** $p < 0.001$.

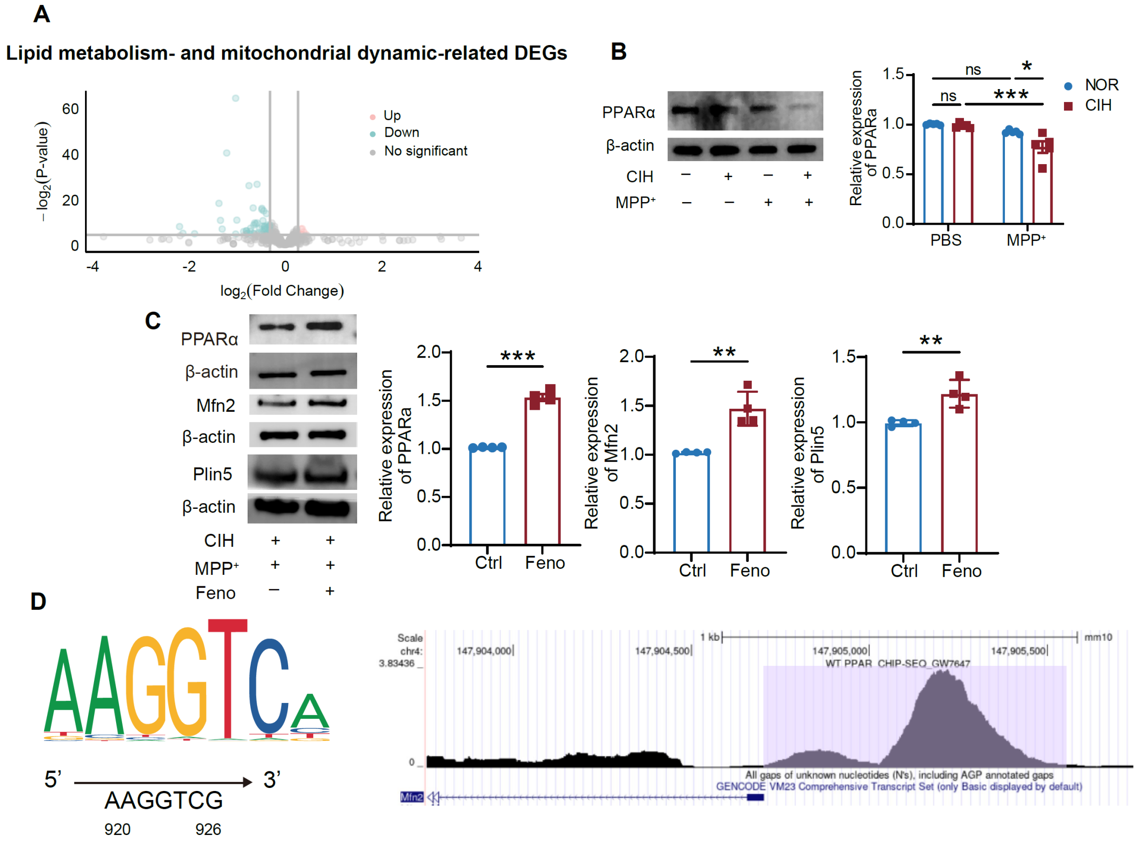

Matrix ID	Name	Score	Relative Score	Sequence ID	Start	End	Strand	Predicted Sequence
MA2338.1	MA2338.1. Ppara	9.446939	0.91825646	NC_000070.7: c147991527-147989527	920	926	+	AAGGTCG

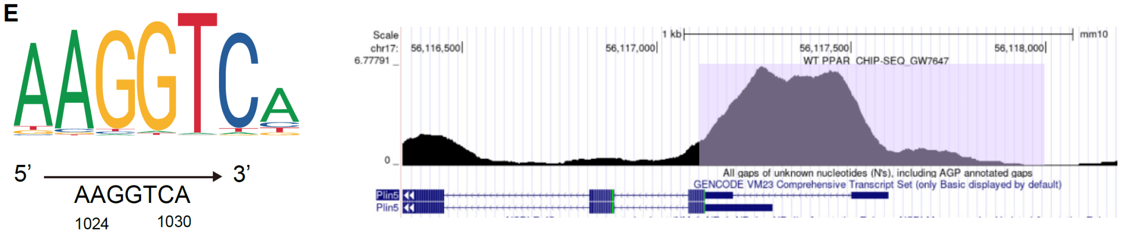

Matrix ID	Name	Score	Relative Score	Sequence ID	Start	End	Strand	Predicted Sequence
MA2338.1	MA2338.1. Ppara	12.915216	1.0	NC_000083.7: c56426606-56424606	1024	1030	+	AAGGTCA

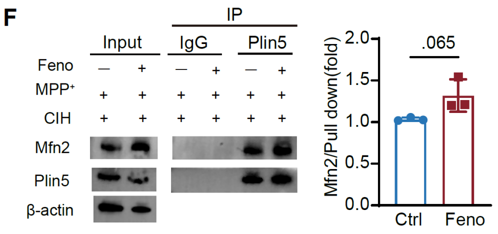

Figure S10 Fenofibrate elevates PPARα, Mfn2, and Plin5 expression. **(A)** Volcano plot of lipid

metabolism- and mitochondrial dynamic-related DEGs between NOR+sMPTP and CIH+sMPTP-treated mice. n = 3 mice in each group. **(B)** Expression of PPARα in MN9D cells in different groups. n = 5 independent experiments. **(C)** Expression of PPARα, Mfn2, and Plin5 in MN9D cells in different groups. n = 4 independent experiments in each group. (**D**) The JASPAR and UCSC database were used to predict the presence of PPARA binding motifs in the promotor region of Mfn2. (**E**). The JASPAR and UCSC database were used to predict the presence of PPARA binding motifs in the promotor region of Plin5. **(F)** Co-IP assay using Plin5 antibody to determine the change of Mfn2 and Plin5 binding in MN9D cells of different groups. n = 3 independent experiments. Two-tailed Student's t-test for all comparisons. ns = no significant, * $p < 0.05$, ** $p < 0.01$, *** $p < 0.001$.

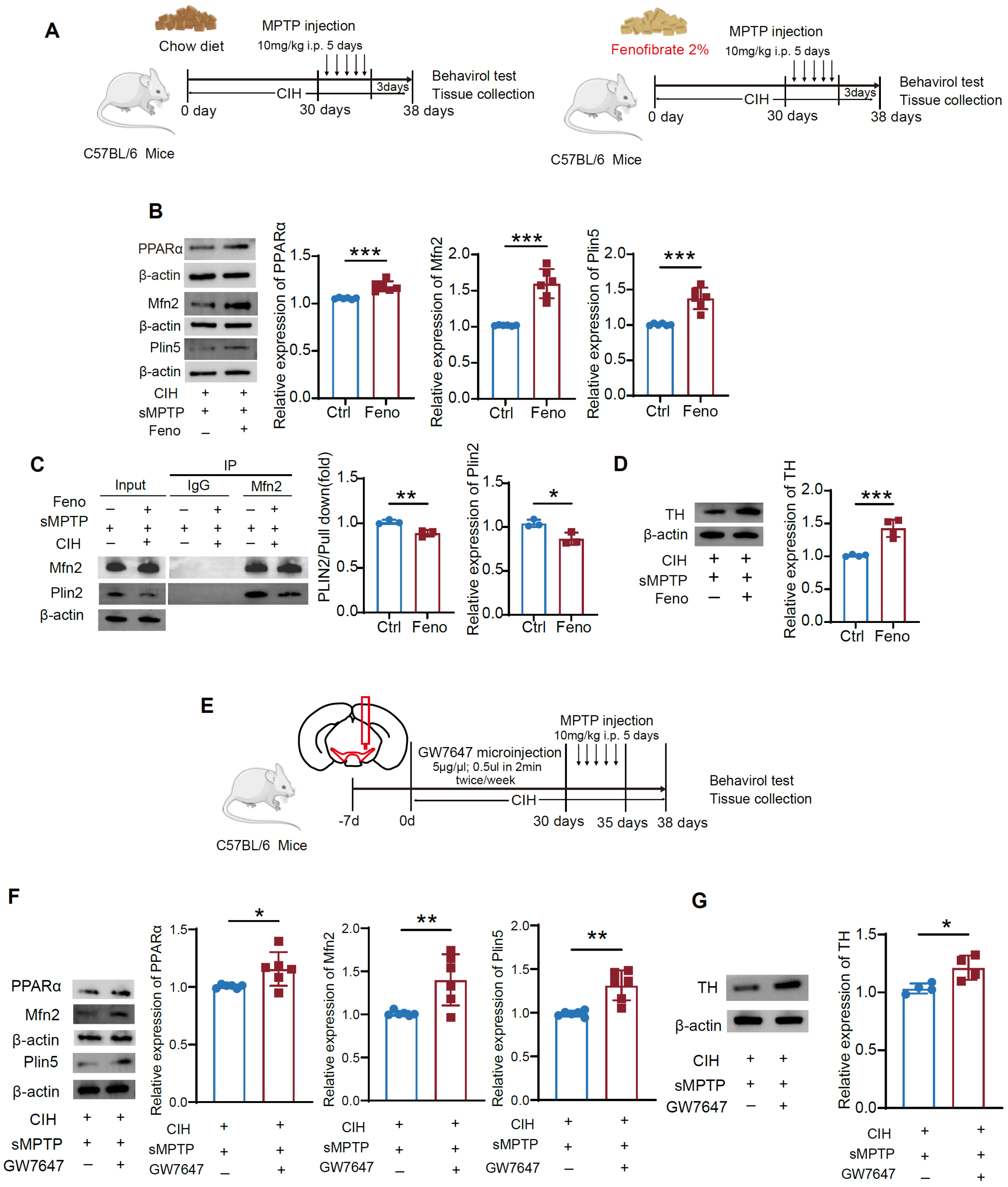

Figure S11 PPARα activation upregulates the expression of Mfn2, Plin5, and TH in SN. **(A)** Schematic diagram of fenofibrate administration protocol. **(B)** Expression of PPARα, Mfn2, and Plin5 in SN of different groups. n = 6 mice for each group. **(C)** Co-IP assay using Mfn2 antibody to determine the change of Mfn2 and Plin2 binding in SN of different groups. n = 3 independent experiments. **(D)** Expression of TH in striatum of different groups. n = 4 mice. **(E)** Schematic diagram of GW7647 microinjection protocol. **(F)** Expression of PPARα, Mfn2, and Plin5 in SN of different groups. n = 6 mice. **(G)** Expression of TH in

striatum of different groups. n = 4 mice per group. Two-tailed Student's t-test for all comparisons. ns = no significant, * $p < 0.05$, ** $p < 0.01$, *** $p < 0.001$.
